# Physical Literacy and Physical Activity of Young Children with Developmental Disabilities: A Scoping Review

**DOI:** 10.3390/children13040548

**Published:** 2026-04-15

**Authors:** Stéphanie Girard, Jason D’Amours, Jessica Bélisle, Annabelle Ross, Annie Paquet

**Affiliations:** 1 Department of Physical Activity Sciences, Université du Québec à Trois-Rivières, Trois-Rivières, QC G9A 5H7, Canada; jessica.belisle@uqtr.ca; 2 Department of Psychology, Université du Québec à Trois-Rivières, Trois-Rivières, QC G9A 5H7, Canada; jason.damours@uqtr.ca (J.D.); annabelle.ross@uqtr.ca (A.R.); 3 Department of Psychoeducation and Social Work, Université du Québec à Trois-Rivières, Trois-Rivières, QC G9A 5H7, Canada; annie.paquet@uqtr.ca

**Keywords:** inclusion, motor development, infants, preschoolers, developmental delays, parents

## Abstract

**Highlights:**

**What are the main findings?**

- Individualized approaches are essential for promoting physical activity participation among children with developmental disabilities.
- Parental involvement is crucial for physical literacy development of children with developmental disabilities.

**What are the implications of the main findings?**

- Design programs and interventions that offer flexible, tailored strategies to accommodate each child’s unique abilities, preferences, and support needs.
- Provide early social support to parents and strengthen their sense of competence, ensuring that individualized approaches extend to the family context.

**Abstract:**

**Background:** Developing physical literacy in children with developmental disabilities (DDs) is essential to fostering their participation in physical activity. According to the Canadian Framework, physical literacy encompasses multiple interrelated components (behavioral, physical, affective, and cognitive). Such engagement provides numerous benefits, including reduced symptoms of anxiety and depression, as well as improved functional and cognitive health. However, children with DD appear to be less active than those without such conditions. Since individuals who are active during childhood and adolescence are more likely to remain active during adulthood, it becomes crucial to better understand how to support the physical literacy development of children with DD, hence enhancing their participation in physical activity. In addition, children with DD remain underrepresented in the literature, particularly with regard to their opportunities to develop their physical literacy and their varied needs, such as limited physical activity options. **Objective:** The aim of this scoping review was to identify and analyze the existing literature on the development of physical literacy and physical activity participation in young children (0–6 years) with DD. **Methods:** Four databases were searched (PsycInfo: *n* = 722; MEDLINE: *n* = 997; ERIC: *n* = 514; CINAHL: *n* = 771), and 25 articles were retained. Characteristics of these studies were analyzed quantitatively, while their scope was analyzed according to physical literacy components. **Results:** Most studies (80%) used a quantitative method, and nearly half (44%) concerned young children with autism spectrum disorder. A little more than half of the studies (52%) focused on early intervention programs. In regard to the scope of the studies, none addressed the cognitive component of physical literacy, indicating a lack in the current literature, and more than half provided information on how to support the affective component. Moreover, information regarding parents’ involvement in physical activity of children with DD emerged from six studies analyzed. **Conclusions:** The results yield interesting insights on how to support the physical literacy development of children with DD and the factors likely to influence their physical activity participation. Early intervention programs promoting physical literacy could be promising avenues to support lifelong physical activity habits for these children.

## 1. Introduction

Increased prevalence of sedentary behaviors in children and their related health disorders, as well as decreased level of physical activity among children [1,2], is detrimental to their overall quality of life [3]. Based on the most recent data available [4], and according to the Canadian 24-h Movement guidelines, 38.2% of preschool-aged children do not meet the recommendation for daily physical activity and 75.6% do not meet the screen time recommendation [5]. In fact, only 12.7% of these children meet the overall national guidelines for physical activity, sedentary behavior and sleep [5]. A similar proportion is observed worldwide: only 11% of preschool-aged children meet physical activity recommendations [6]. These guidelines suggest at least 180 min of varied physical activity throughout the day, with one-third involving energetic play [7]. Among children and youth (5–17), only 39% meet the recommendations of 60 min of moderate to vigorous activity per day [4,7]. As for children with disabilities and medical problems, they appear to be less active than those without such conditions [8,9,10].

Early childhood is a crucial phase because it is decisive for the adoption of healthy lifestyle habits [11,12]. Indeed, inactivity during childhood is associated with several health problems in adulthood (e.g., diabetes, high blood pressure, heart disease, asthma, arthritis), while participation in regular physical activity during youth can significantly prevent chronic diseases later in life [13,14]. For young children, physical activity shows positive effects for health at the physical level but also at the social, cognitive and affective levels [15,16], notably by reducing anxiety and depression symptoms and improving social-emotional learning, social cognition and general mental well-being [17,18,19]. Specifically, daily physical activity is characterized by improvements in several areas such as bone and skeletal health, motor skill development (e.g., running, jumping, swimming), psychosocial health (e.g., well-being, motivation, enjoyment), cognitive development (e.g., language development, executive function, attention), and social behaviors (e.g., self-esteem, prosocial behavior) [20,21,22]. In preschool-aged children, results of a recent systematic review indicated that meeting physical activity guidelines was strongly associated with benefits such as emotional regulation, motor skills, social behaviors, and cognitive development [23]. Physical inactivity and sedentary behaviors, on the other hand, has major negative effects on several areas of a child’s development [24,25], as well as their physical literacy development [26], which can affect their functioning, overall well-being, and quality of life [27]. For example, physical activity practice can affect basic body management skills such as stability, the ability to stop a movement or object at a certain time, spatial awareness or the simple ability to recognize certain parts of the body [28]. A lack of fundamental movement skills can lead to insecurity and low self-esteem at the start of school when children compare themselves to those who perform well in these areas [24,29]. This early disparity predisposes children to a higher risk of obesity, metabolic disorders, poorer general health, increased social isolation and a lower quality of life due to sedentary behavior [25].

### 1.1. Physical Activity of Children with Developmental Disabilities

Despite the potential benefits of physical activity for young children on their well-being and quality of life [30], there is a discrepancy between children with and without developmental disabilities (DDs) [31]. According to the American Psychological Association [32] (para. 1), developmental disability is defined as “a developmental level or status that is attributable to a cognitive or physical impairment, or both, originating before the age of 22. Such an impairment is likely to continue indefinitely and results in substantial functional or adaptive limitations.” Research shows that people with a disability (e.g., autism spectrum disorder (ASD), learning disorders, developmental coordination disorder, communication disorders, cerebral palsy, etc.) are less likely to participate in physical activity, more sedentary, usually less fit [33], and most likely to develop anxiety and depression symptoms, as well as a low self-esteem [34,35]. However, the benefits of physical activity for children with and without DD are similar. For example, physical activity has been associated with the development of socialization, interpersonal skills, and motor abilities in children with ASD [36]. Similarly, for children with physical disabilities, participation in physical activity can enhance social connection, promote peer acceptance, and create opportunities for friendship [10]. Given the many benefits of physical activity for all children, future research would do well to explore effective ways to promote children with DD participation. For instance, physical literacy is an important factor in all children’s development because it enhances knowledge and behaviors related to the daily practice of physical activity and provides effective tools in this regard [37].

### 1.2. Physical Literacy: An Active Ingredient for an Active Lifestyle

Physical literacy is defined as “the motivation, confidence, physical competence, knowledge and understanding to value and take responsibility for maintaining purposeful physical pursuits/activities throughout the life course” [38] (p. 29). As the definition indicates, physical literacy includes behavioral, physical, affective and cognitive components [27]. The behavioral component refers to voluntary engagement in physical activities [27]. The physical component involves the development of physical competence to develop and perform movement skills, create certain patterns, and experience multiple movement intensities and durations [27]. These abilities allow participants to join a wider range of physical activity settings. The affective component refers to the individual’s motivation and confidence to take part in physical activities [27]. In other words, it refers to enjoyment and the self-belief in one’s ability to participate and adopt multiple physical activities as part of life. The cognitive component—knowledge and understanding—involves the ability to qualify, identify and express the impact of movements on health and an active lifestyle through safety features regarding physical activity and multiple settings and environments [27]. Developing all physical literacy components as a whole should lead to a lifetime engagement in physical activity [38], hence having more opportunities to develop a quality of life and well-being [39]. Among the several frameworks that have been developed to define physical literacy’s components, this article focused on the initial Canadian consensus framework, as depicted in Table 1. This framework was selected because it is grounded in a holistic and developmentally oriented conceptualization of physical literacy, integrating physical, affective, cognitive, and behavioral domains [40,41]. It has also been widely operationalized in applied and early childhood contexts, particularly within Canadian education and community settings [40]. Importantly, this framework aligns with multidimensional constructs relevant to children aged 0–6 years and represents one of the few approaches that has been translated into observable and measurable indicators suitable for young populations [26,42].

According to Whitehead [44], the physical literacy journey passes through six different age-related stages: (1) preschool years; (2) foundation/early and primary school years; (3) secondary school years; (4) early adulthood years; (5) older adult years; and (6) adult years. The first stage is critical for the foundation of physical literacy as regards the development of fundamental movement skills, because these skills are the building blocks for the practice of physical activity and are essential to sustain children’s motivation and competence: deprivation of movement during this stage can be devastating for children’s development [45]. Indeed, Cairney and colleagues [26] argue that physical literacy promotes positive physiological adaptations to stress, leading to two key effects on mental and social health: reduced risk of depression and anxiety, and increased self-esteem. Thus, during the early years, all adults (e.g., parents, childcare staff, camp counsellors, recreation practitioners, preschool teachers) in a child’s different environments (e.g., home, childcare, leisure or community settings, and physical education) should work with the aim of promoting and encouraging physical activity participation [44]. Indeed, developing children’s physical literacy promotes their physical activity participation, which, in turn, develops their physical literacy [46,47]. As suggested by López-Gajardo and colleagues [48], physical activity is a component of well-being that should be given greater attention.

In keeping with Whitehead’s conceptualization, the physical literacy concept is inclusive in itself: “all can make progress on their physical literacy journey, the challenge being to capitalize on individual potential to take part in whatever physical activity is within an individual’s capacity” [38] (p. 31). Results of a scoping review focusing on children 6–12 years with behavioral and mental health disorders indicated that they were less physically literate than children without these conditions, and that they may need more support and individualized approaches to support their development of physical literacy [49]. Similarly, considering that living with a DD can lead children to have emotional and social issues, as well as low self-esteem [29], children with DD are more likely to experience isolation, bullying and discrimination, which has a direct impact on their mental health [47,50]. Accordingly, more evidence-based data are needed to better understand how to support the development of physical literacy and the physical activity practice of young children (0–6 years) with DD [20,51]. Indeed, the 2024 Children & Youth Report Card highlights the fact that “research that explores strategies for adapting physical literacy practices is required to better align physical literacy research with the needs of children with disabilities and medical conditions” [4] (p. 53).

### 1.3. Study Rationale

According to Cairney and colleagues [47], physical literacy is a determinant of health. However, the development of physical literacy is often a challenge for children with DD. Fortnum and colleagues [52] also argue that the model by Cairney and colleagues [26] has not been sufficiently tested in certain populations, even though the proposed model aims to be inclusive. Indeed, few programs for young people living with disabilities have been explored [10]. Fortnum and colleagues [52] therefore recommend conducting research on specific groups (e.g., children with developmental coordination disorder). In their systematic review, Carl and colleagues [53] found that, for people going from six years old to adulthood, physical literacy interventions have significant effects on all components of physical literacy, with a strongest effect for physical outcomes. In addition, in their study with young neurotypical children (aged 3–5 years), Li and colleagues [54] argue that a better understanding of the correlations between the components of physical literacy is needed to guide physical literacy interventions in the early ages. Recently, it was found that programs using physical activity have positive effects on children living with autism spectrum disorder, particularly on improving executive functions enhancing brain functions [55], reducing repetitive behaviors and altering triple network dynamic connectivity [56]. Building on these promising results, and addressing the gaps related to physical literacy development in early childhood for the population of children with DD, the aim of this scoping review was to identify and analyze the existing literature on the physical literacy development and physical activity participation in young children (0–6 years) with DD. This age range corresponds to the “Active Start” stage of the Long-Term Development in Sport and Physical Activity Framework, which includes the early development of physical literacy [40].

## 2. Method

A scoping review allows for the identification of knowledge gaps and future research perspectives and provides an overview of the number and type of studies on a specific subject [57]. In keeping with the framework of Arksey and O’Malley [58], the review consisted of five steps, namely: (1) identifying the research objective; (2) identifying relevant studies; (3) study selection; (4) charting data; and (5) summarizing and reporting results. This review was conducted in accordance with the PRISMA extension for scoping reviews (PRISMA-ScR; see Supplementary Materials Table S1).

### 2.1. Studies Identification

The databases used were APA PsycInfo (722), MEDLINE (997), ERIC (514), CINAHL (771). These databases were selected based on the recommendations of a specialized librarian, who also assisted in generating the keywords for the following categories: (1) inclusion, (2) developmental disabilities, (3) physical literacy, (4) physical activity, (5) children. The keywords used for the current search of English-language databases were (inclusion OR “special need*” OR autism* OR “developmental disorder*” OR “intellectual disability” OR adapted OR “developmental disabilit*” OR “developmental delay” OR “autism spectrum disorders” OR “adaptive behavior measures” OR “intellectual development disorder” OR “delayed development”) AND (“physical literacy” OR adequacy OR confidence OR enjoyment OR motivation OR “perceived competence” OR “self-efficacy” OR predilection OR knowledge OR understanding OR “self-perception” OR “self-concept” OR “self-esteem*” OR affectiv* OR cognitive) AND (“physical activit*” OR “active play” OR exercise OR recreation OR “recreational activit*” OR “day camp” OR “summer camp” OR leisure OR “physical activity experiences”) AND child* (see Supplementary Materials Table S2 for full search strategy).

### 2.2. Studies Selection

After relevant studies were identified, the selection criteria presented in Table 2 were applied for population study, year of publication, type of study, context and language.

**Figure 1:**
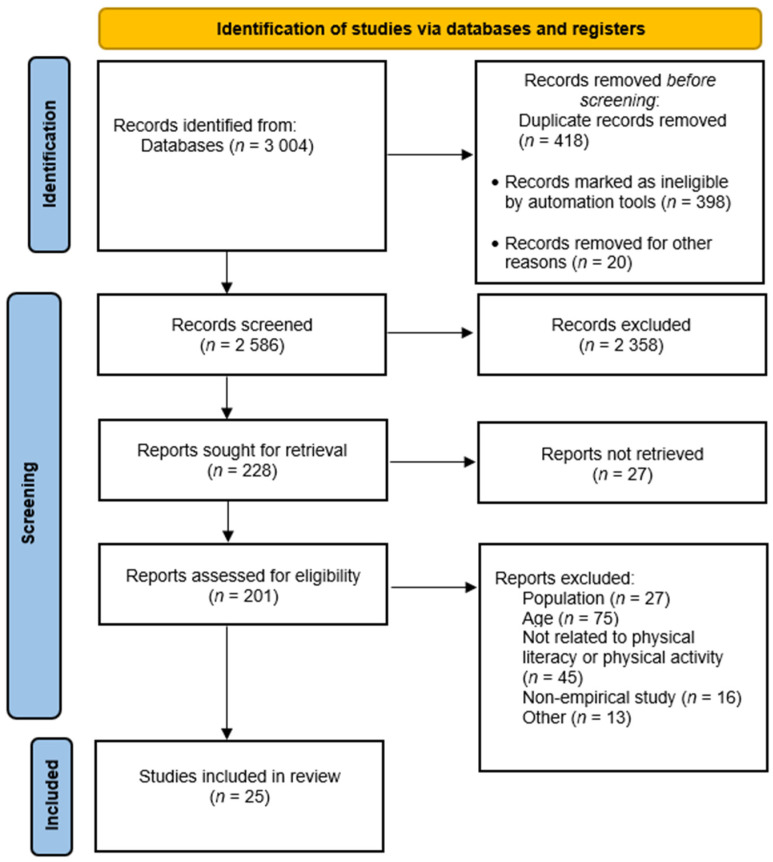
PRISMA diagram.

Two of the five authors reviewed the titles and abstracts of all 3004 articles and selected the articles individually. They then pooled the results of their selection, making sure to obtain the same results. Although inter-rater reliability was not formally assessed, a high level of agreement was observed between reviewers and verified through comparison and discussion. If doubts persisted about certain articles, a third author reviewed the article and made the final decision; 2358 articles were excluded because of the population, context or nature of the study. Following a complete reading of the article, 176 articles were excluded, and 25 were selected for the final analysis. The Prisma diagram [59,60] presented in Figure 1 summarizes the process and results of the study identification and selection.

### 2.3. Charting Data

Data from the 25 articles retained (see Supplementary Materials Table S3) were extracted using an analysis grid including the study aim, participants, data collection and analysis, main results, and conclusion. This grid allowed us to identify the study characteristics (nature of the studies; see Supplementary Materials Table S4) and main themes (scope of the studies) offering information on the physical literacy development and physical activity of children with DD. Relevant results from each study was coded and then classified according to physical literacy components: (1) behavioral; (2) physical; (3) cognitive, and (4) affective, allowing for the systematic grouping of relevant findings and facilitating comparisons across sources.

### 2.4. Summarizing Results

Study characteristics (year of publication, country, methodological approach, number of participants, and type of disability) were analyzed using descriptive statistics. To analyze the scope of the studies, a deductive thematic analysis was conducted to identify the recurrent information (codes) and highlight patterns that consistently emerged within the included sources according to the components of physical literacy. This synthesis also enabled the identification of emerging trends, revealing areas of underexplored interest in scientific literature.

In sum, data analysis was conducted following a five-step process informed by thematic analysis principles [61] within a scoping review approach [58]: (1) familiarization with data (all 25 articles were read in full); (2) initial coding (relevant data was extracted and coded based on the review question); (3) grouping of codes into categories (organization of the extracted data to facilitate comparison across sources); (4) grouping of categories into themes; (5) revision, synthesis and reporting (themes were summarized to map the extent of the literature, highlight knowledge and inform future research directions).

## 3. Results

Table 3 indicates how many articles were published each year since 2010. It can be noted that, after a greater number of publications in 2017, very few publications have been made in the last decade. 

More than half the studies were conducted in the United States (*n* = 13/25). Two were conducted in Canada and two in Israel. The United Kingdom, Germany, Switzerland, Greece, India, Taiwan, Japan and Spain accounted for one study each. Additionally, the vast majority of studies (*n* = 20/25) used a quantitative method, while only five used a mixed approach [62,63,64,65,66].

The number of participants per study varies from three children (in three studies; [64,67,68]) to 18,818 [69] (*M* = 9427; *SD* = 13,280.88). The majority of studies considered children with autism spectrum disorder (ASD; *n* = 11/25), followed by children with general DD (e.g., developmental delay, gross and fine motor skills delay; *n* = 6/25) and a developmental coordination disorder (DCD; *n* = 4/25). One study considered children with intellectual disability (ID; *n* = 1/25), children with Down syndrome (DS; *n* = 1/25), children with attention-deficit/hyperactivity disorder (ADHD; *n* = 1/25) and children with several diagnoses (developmental disorder, ASD, communication disorder, ID and other; *n* = 1/25).

In total, 13 studies focused on programs for children with DD. Details in this regard are provided in Supplementary Materials Table S5 and include: (1) aim, (2) duration, (3) schedule, (4) facilitators, (5) activities, and (6) assessment of fidelity of implementation. Duration of programs varied from three weeks to eight months, with activities scheduled one to five days a week and lasting 10 min to four hours per day. Information regarding the assessment of fidelity of implementation was reported for a little more than half of the programs (*n* = 7/13).

As no study has addressed the cognitive component of physical literacy, data was classified according to three physical literacy components: (1) behavioral (*n* = 5 studies); (2) physical (*n* = 10); and (3) affective (*n* = 14). Moreover, information regarding parents’ involvement in physical activity of children with DD emerged from six studies analyzed. Next sections present the summarized results.

### 3.1. Behavioral Component

Based on two studies, results indicated that children with ASD tended to meet the recommended 60 min of moderate-to-vigorous physical activity per day [70,71]. However, results also indicated that they exceeded the time spent on sedentary behaviors, and that participation in an early motor skill intervention did not increase levels of physical activity. In fact, children with ASD spent most of their day (about 8 h) in sedentary activity [70]. In addition, two studies observed a positive association between motor ability and participation in moderate-to-vigorous physical activity as well as sport club attendance, whereas motor delays were negatively associated with these behaviors [63,69]. In contrast, sedentary behaviors, such as TV viewing, were positively associated with motor delay [69].

According to the results of three studies using observations [66,67,72], sedentary behaviors and engagement in physical activity of children with DD seems to vary depending on the context of practice, the type of grouping, and the motivational climate. One study indicated that children with DD were more physically active outdoors than indoors, and that they were more active in open spaces and while playing with portable equipment, such as balls, than in fixed playgrounds [72]. However, the opposite pattern was observed for children with ASD, for whom the highest levels of moderate to vigorous physical activity were observed during indoor sessions, which may be explained by the fact that their behavioral responses appeared to be more variable outdoors, likely because activities and settings differed [67]. In indoors environments, although children with DD spent most of their time sedentary (81.5%), they were more likely to be physically active during therapy sessions than during group activities. They were also more physically active in solitary or one-on-one contexts compared with adult-supervised group activities [72]. One study further noted that creating a mastery motivational climate flexible enough to enable children with lower skills to experience success enhanced children’s engagement, while emphasizing that time was required for this positive effect to become evident [66].

### 3.2. Physical Component

According to the results of five studies, children with DD exhibited lower motor skills and motor competency than children without DD. Specifically, children diagnosed with ASD exhibited significantly lower gross, fine and total motor skills than their peers with typical development (TD) [71], and ASD characteristics (social affect and restrictive repetitive behaviors) were negatively associated with fundamental motor skills competency [73]. Compared with children with TD, children diagnosed with DCD display markedly lower levels of motor performance evaluations, lower motor skills and lower processing skills [63,74]. Motor competence was also lower in children with ADHD compared with TD children, but both improved similarly during the inclusive group psychomotor therapy program [75].

In total, nine programs implemented in different contexts (e.g., during summer, at preschool, at a daycare center, by specialized services) focused on improving fundamental motor skills development or motor competence of children with DD [62,65,66,70,73,75,76,77,78], and three of them were evaluated using a mixed-methods design [62,65,66]. They proved effective in terms of progress in balance, locomotion, coordination, object control (e.g., ball skills), single leg stance and grip strength, movement skills and motor competence for children with DD. One program employed an ABA design to develop, notably, fine and gross motor skills for children diagnosed with ASD and showed promising results: both types of motor skills increased significantly, and parents reported maximum improvements in gross motor skills (e.g., throwing a ball, running, climbing stairs, jumping) [79].

### 3.3. Affective Component

Four studies provide information regarding the enjoyment of children with DD during participation in physical activity from their parents’ point of view. Results of two studies indicated that children diagnosed with DCD or ASD recruited in clinics had less fun during physical activities, leisure time or social interaction because of self-consciousness about their deficits in areas such as motor skills, and this shyness affects functioning [80,81]. Nevertheless, in the context of play in preschool, results of two other studies comparing children with probable DCD and children with TD, they seemed to have similar physical and emotional well-being, and both had high levels of enjoyment during outside-school activities [63,82]. One study conducted in preschool using a quasi-experimental design also reported that more time spent watching other children play was related to higher well-being for children with probable DCD [82].

Six studies conducted in different settings (e.g., clinical, preschool, university) employed diverse methodological approaches, including cross-sectional, single-case (multielement), quasi-experimental, and single-blind randomised controlled designs to highlight that children with DD participation in physical activity can be encouraged by considering their motives or preferences. Indeed, a preference for active physical activities revealed a positive association with motor ability (e.g., manual dexterity, aiming, catching and balance), and the higher the preference for an activity, the higher the level of enjoyment of the children diagnosed with DCD [63]. The fact that children with DCD did not report reduced enjoyment of their activities implies that, although they displayed lower participation diversity, their overall level of enjoyment remained similar to peers with TD [63]. To motivate young children with DD to be physically active, the Scratch 2.0 [61] are low-cost, commercially available and effective methods because they are easily adapted to each child’s interests and preferences [64,76,77]. Indeed, an increase in physical activity was observed in studies using these types of support [64,77] and seems to encourage them to work harder [76]. For children diagnosed with ASD, another low-cost strategy to increase physical activity would be the use of photographic activity schedules (i.e., pictures in a book demonstrating the physical activity such as running, skipping, hopping, or crawling) requiring only short teaching, generalization, and maintenance (without using prompts) phases [67,73]. As for the more “traditional” gym session, it seemed to be more psychologically challenging for children with ASD: they were less motivated to do the exercises, and that, even if an increase in physical activity occurred [76].

Three programs for children diagnosed with ASD were used to promote social interaction, communication and motivation through participation in a physical activity (e.g., aquatic, dance and outdoor activities) [68,83,84], while one inclusive group psychomotor therapy program aimed to improve socio-emotional competence of children with and without ADHD [75]. These programs benefited these children insofar as they offered opportunities to enhance social interaction and communication skills and were mainly implemented in community-based or group settings, thereby encouraging these children to participate still further [68,75,84]. Furthermore, one such program took place in an aquatic setting, which is a motivating and sensory-rich environment for children diagnosed with ASD to socially interact with caregivers and friends [83]. Dance activities also appear motivating for children with DD and can easily be incorporated in childcare centers and preschools settings. Indeed, they resulted in more complex and interactive play within preschool learning centers using priming material only [68]. Moreover, taking part in a Dance Movement Therapy intervention helped children with ID decrease affective problems. However, without a research team present, preschool teachers may require training and support to add dance activities to their daily routine [78].

Five programs [68,70,73,83,84] were primarily oriented around group physical activities for children diagnosed with ASD, but were also intended as a good complementary approach to the traditional interventions for this population. More precisely, these studies advanced the idea of using the physical activity playgroup as part of a comprehensive early intervention because this strategy supports playfulness and participation in community settings. In sum, activities allowing for social interaction with peers and leisure time enjoyment seemed to be a motivating factor for children with DD [83].

### 3.4. Parental Involvement in Physical Activity for Children with Developmental Disabilities

Two studies reported information regarding parental perceptions and satisfaction. Specifically, parents’ perceptions about their child’s physical activity were significantly correlated with delays in overall adaptive functioning, daily living skills, socialization, as well as motor skills [81], and it appears that parents of children diagnosed with DCD were frequently less satisfied with their child’s level of participation [74]. In fact, parents were less likely to view physical activity as beneficial, as they reported more barriers and expressed lower confidence in their child’s ability to participate effectively [81].

Four studies focused on perceived barriers from the parents’ point of view. Although parents may be willing to offer their child opportunities for physical activity, they encounter barriers such as child behaviors (e.g., lack of interest or motivation, difficulty understanding and following directions, or sensory issues), environmental factors (e.g., lack of community-based resources or safe place to practice physical activity, time limitations, etc.) or low levels of self-efficacy [65,85,86]. This is particularly consequential given that children with DD are highly dependent on their parents and typically spend most of their time with them, which can affect their opportunities for participating in physical activities [74,85], particularly when parents report difficulty finding time in their busy schedules [65]. This is especially true considering that parents who perceive fewer barriers to PA report a higher level of physical activity by their children diagnosed with ASD [81]. Moreover, parents’ perception of barriers, self-efficacy, and knowledge and ability to perform a home exercise program was associated with the frequency and duration of implementing the program [86].

Two studies provide recommendations to encounter these barriers, such as providing social support [65,86]. To do so, using a web-based parent-mediated fundamental motor skill intervention (Project SKIP; using video examples, providing reminders, instruction, and guidance) via a private Facebook group was recognized as highly feasible, effective, and motivating. However, parents would still have preferred to have had the opportunity to meet the other families involved in the intervention [65]. In addition to being able to recognize parents who have a low sense of self-efficacy, health professionals could apply multiple motivational strategies, such as providing information about the child’s progress, justifying the usefulness of exercises, giving advice to insert the home exercise program into the daily life, maintaining frequent contact with parents in order to review their skills, and give feedback about their performance could facilitate adherence to frequency [86].

## 4. Discussion

This scoping review synthesized the current state of evidence regarding the development of physical literacy and physical activity participation among children with DD aged 0–6 years. Over the past decade, the number of publications addressing this topic has remained limited, with only few studies published each year. Although over half of the included studies originated from the United States, the remaining articles were dispersed across multiple countries, reducing the ability to draw contextually grounded or culturally specific conclusions. The representation of DD was also uneven: nearly half of the studies focused exclusively on children with ASD, whereas the others included heterogeneous DD groups, reflecting research patterns with older children [87,88]. This higher representation may be explained by the increasing prevalence of those type of diagnosis in children [89,90]. Nevertheless, this means that other types of DD remain underrepresented. Studies that include children with a variety of DD could certainly be more valuable to enrich our understanding of these children’s needs in terms of their physical literacy development and physical activity participation, as suggested by D’Amours et al. [88,91].

Methodologically, despite one notably large sample [69], some sample sizes were quite small [64,66,67,68,83], which restricts external validity. Furthermore, most studies adopted quantitative designs, and a little more than half involved program evaluations whose characteristics varied considerably (See Supplementary Materials Table S5). This substantial methodological heterogeneity, combined with the overall scarcity of evidence, limits the generalizability of findings and calls for caution in interpreting emerging patterns. Regarding studies’ findings, overall, they addressed three components of physical literacy—behavioral, physical, and affective—as well as children with DD’s parental involvement. Yet, important gaps remain in how these variables are operationalized in early childhood among children with DD.

Unfortunately, our results did not provide information on the cognitive component of physical literacy, which refers to individuals’ knowledge and understanding of physical activity practice. Given the young age of the population under study, this aspect may seem less of a priority for research [92]. Indeed, the cognitive component appears to be the least studied component across research regarding youth with disabilities [93,94]. Nevertheless, even young children with DD require certain kinds of knowledge such as the ability to recognize signs of fatigue and to respect their limits. For instance, in preschools, children should develop healthy lifestyle habits by exploring the world of food, learning different ways to relax, acquiring hygiene practices and developing safety awareness [95]. In the childcare setting, similar knowledge must also be introduced according to educational programs, which emphasize the importance of supporting children’s physical development by creating an environment favoring the adoption of healthy lifestyle habits [96,97]. Therefore, future research should focus on better operationalizing and assessing the cognitive component of physical literacy in early childhood among children with DD.

At first glance, results pertaining to physical activity practice are fairly encouraging, given that children with ASD spent as much or more time practicing than their peers with TD [70,71], which meets the Canadian guidelines of 60 min per day for children between 5 and 17 years old. However, in early childhood, these guidelines recommend to engage in at least 180 min of varied physical activity spread throughout the day, while favoring energetic play [7]. Moreover, being physically active is not sufficient to counter the harmful effects of sedentary behaviors as both habits can coexist [98,99]. In consequence, results are less optimistic with respect to sedentary behaviors, for which children with ASD exceeded the recommended time [70,71]. These studies were conducted exclusively with children with ASD; further research is needed to determine whether these findings extend to children with other types of DD.

With regard to practice contexts, in preschool, children with DD tended to be far more physically active outdoors than indoors and that children were indoors 79.6% of the time, which limited their exposure to environments and open spaces where they were usually more active [72]. In contrast, children with ASD were more physically active indoors than outdoors [67]. The authors argued that interventions should be individualized. Taken altogether, these observations highlight the importance of tailoring intervention contexts to individual needs (i.e., using an individualized approach), rather than applying the same environment, grouping format, or tasks to all participants [67,73], which is consistent with results regarding physical literacy development of children (6–12 years) with behavioral and mental health disorders [49]. Fortunately, it appears that interventions emphasizing physical activity practice among preschoolers increase physical activity practice among toddlers, particularly when facilitators are well trained [100]. Indeed, the results of a recent scoping review [101] on the participation of children diagnosed with ASD in summer camps specify that this environment favors the development of their physical literacy, and that trained practitioners are essential for this purpose.

Although our results indicate that children with DD display lower motor skills and motor performance than children without DD [63,71,74,75], it is encouraging to note that both can improve similarly during psychomotor intervention [75]. Results of studies including youth with DD (5–24 years old) indicated that, no matter the type of DD, improvements are possible [88,91]. Indeed, the implementation of effective programs aimed at developing children’s motor skills represents a promising avenue of research. Among the selected studies, programs showed encouraging results for enhancing the gross and fine motor skills of young children with DD by means of an intervention implemented during summer [65,70], at preschool [62,76,78], at a daycare center [66] or by specialized services [73,75,77,79]. These results are consistent with those of a recent meta-analysis focusing on healthy preschoolers [102].

Generally, scientific literature indicates that the physical component of physical literacy is typically the most frequently examined among youth with disabilities [92,94,103]. Interestingly, our review shows that, in research involving younger children with DD, the affective component appears to be more prominently represented. This finding is noteworthy, as it highlights the perceived importance of supporting young children’s motivation, enjoyment and play during physical activity participation and play. Indeed, becoming physically active often starts with the motivation to do so, which highlights the need to consider the interplay among the components of physical literacy. In this regard, some authors have sought to illustrate these reciprocal relationships by integrating concepts from self-determination theory into the physical literacy cycle [47,104,105,106]. Specifically, fostering children’s motivation—through the satisfaction of autonomy, competence and relatedness—promotes active participation, which in turn enhances movement competence and self-confidence, thereby further reinforcing motivation. According to these authors, this cycle also operates in the opposite direction, in that experiencing positive challenges aligned with children’s abilities, enjoyment and positive relationships during active participation fosters motivation, while self-confidence contributes to the development of movement competence. In line with our findings regarding the absence of the cognitive component in the retained articles, additional research is warranted to further clarify how this component can be incorporated into the cycle.

According to our results, it remains unclear whether children with DD enjoy physical activity as much as children without DD, as some studies suggest they do not [80,81], while others indicate they do [63,82]. However, it should be noted that these results are based on parents’ perceptions: it is therefore possible that they reflect their own concerns rather than those of their children. Nevertheless, parental perceptions need to be considered, as Martin and Choi’s [107] findings show that parents’ perceptions of their children’s enjoyment outweigh motor skill levels in determining their support for sustained physical activity. Fortunately, our results also provide useful information on how to develop the affective component of physical literacy of children with DD and, hence, support their motivation and confidence to engage in physical activities. Specifically, our results reveal the importance of considering young children’s interests or preferences when encouraging them to take part in physical activities across different settings [63,64,76,77]. Toward this end, low-cost commercially available devices such as Scratch 2.0 or the Nintendo Wii^TM^ can easily be adapted to suit each child [64,76,77]. Even using photographic activity schedules (instead of videos) appears sufficient to support children with DD to be physically active in different contexts (e.g., outdoors) [67,73]. In fact, the importance of focusing on children’s interests to support their motivation is a widely recommended motivational strategy in physical education [108,109] and leisure settings [106]. Similarly, establishing a mastery motivational climate is also a promising way to support motivation and engagement in physical activity. In fact, one particular program [66] was based on TARGET principles (task, authority, recognition, grouping, evaluation and time) [110] that have proved effective in increasing children’s level of engagement in gross motor activities. The advice for establishing a mastery motivational climate based on these principles is to work in stations with several levels of challenge, allow children to choose the order of the stations they try, provide individual feedback focused on the child’s personal progress, encourage cooperative learning, recognize effort and improvement, and allow children to spend the time they need at each station [66]. In accordance, creating a mastery motivational climate represents an effective way to tailor interventions to children’s varying abilities and needs according to parents’ point of view.

As indicated in the physical literacy cycle integrating self-determination theory, developing physical literacy of children with DD often depends on their capacity to interact socially, which may present a challenge [10]. According to our results, children with lower motor performance took part in more social activities alone [63]. During indoor activities, for example, preschool children with disabilities were more physically active in solitary (e.g., therapy) or one-on-one group contexts than during group activities supervised by an adult [72]. Consistent with the high-quality inclusive and empowering leisure experience framework [106], the development of positive relationships helps support individuals’ motivation and engagement by nurturing the need for relatedness [111,112]. Thus, providing opportunities for children with DD to forge positive relationships through physical activities while at the same time varying the social composition of the group (with preference given to smaller groups) [72,113,114], should further motivate these children to engage in physical activities.

Accordingly, the ability of other children to include those with disabilities and understand concepts such as inclusion, fairness and equity should not be underestimated [115]. This is especially important given that programs valuing group activities demonstrate positive effects on both the physical and social skills of children with DD [68,70,73,75,78,83,84]. Improving these children’s motor skills, more specifically, resulted in a decrease in solitary play [70]. Furthermore, children with disabilities appear to find leisure group activities such as dancing and aquatic or outdoor activities motivating and fun [68,78,83,84], something that is especially true when their various interests are considered in the animation of these activities. Indeed, performing exaggerated movements to music, imitating one another, and playing together with objects facilitated social interactions between children as well as between children and their various caregivers, further supporting their motivation to be physically active [68,83,84]. In such, interventions such as Dance Movement Therapy [78] are promising avenues to explore in preschools. With a view to making this type of intervention accessible, tools for early childhood staff should be made available. For example, a program using similar principles was recently developed and published “Body language: Experiencing moving stories with children” (Free translation by the author) [116]. However, to implement such programs, preschool teachers may require training and support [78,117].

The scoping review highlights the central role of parents in shaping young children’s physical activity opportunities, a pattern also observed among parents of older children with or without disabilities [118,119,120,121]. In fact, at this age, play opportunities are largely controlled by the parents of children with DD [63]. Considering that delays in socialization and motor skills have had a negative impact on parents’ perceptions of the benefits of physical activity for their children [81], this may lead parents to provide fewer opportunities for their children to engage in physical activity. This review also identified several barriers experienced by families, including individual factors (e.g., low self-efficacy), child-related behaviors, and environmental constraints [65,85,86]. These findings are consistent with barriers commonly reported in the broader literature on physical activity participation among individuals with disabilities [122,123,124,125,126]. Fortunately, the review also identified ways to support parents in this crucial role and, in doing so, strengthen their sense of self-efficacy. For instance, results indicated that community programs and parent-mediated web-based programs can help them learn how to support their child’s participation and understand the importance of establishing family routines involving “active play” [65,83]. Moreover, social support, whether provided by experts [86] or by other families [65], appears to be a critical factor to consider. Although various social media platforms now offer access to virtual communities, families also express a need for in-person interaction. In this regard, the Ministry of Health and Social Services [127], in its 2025–2035 National Health Prevention Strategy, highlights the key role of community workers in supporting families’ adoption of healthy lifestyles from the prenatal period onward, given their optimal positioning to do so. However, research in this area remains scarce.

### 4.1. Limitations of the Review

Certain limitations must be considered when interpreting the results. First, although the focus of the study was on both physical literacy and physical activity, the studies selected did not always explicitly mention their links with physical literacy development. The information retrieved was thus based on how authors conceptualized and interpreted the results with a view to better understanding their implications in terms of the development of physical literacy and the physical activity participation of children with DD. This choice was made to gather as much information as possible. Second, because we did not assess the quality of the selected studies, some results may be based on “poor” evidence. However, the nature of this review does not call for this type of analysis and the inclusion criteria regarding the type of study limited our choice to empirical studies, which allow for a certain level of scientific value. Third, nearly half of the studies (44%) focused on children with ASD whereas other conditions such as ID were underrepresented. Therefore, this imbalance limits the transferability and generalizability of findings to the broader population of children with DD. Fourth, the age range (0–6 years) encompasses substantial developmental variability, and because most studies did not provide age-stratified analyses, it was not possible to determine whether findings differed meaningfully across developmental stages. Finally, the substantial variability in both intervention characteristics and study designs constrains the interpretation of findings regarding the long-term development of physical literacy and sustained physical activity participation in children with DD.

### 4.2. Implications for Practice

When helping families with young children with DD, practitioners should try to remove barriers by providing information on developmentally appropriate expectations and how to organize the environment to offer children and their family positive physical activity experiences without increasing the family’s load [85]. Accordingly, the advice is as follows: (1) provide information regarding the child’s progress, (2) justify the usefulness of the recommended activity, (3) offer advice on how to incorporate the physical activity into the daily routine, (4) support parents’ ability to perform the activity with their child and (5) ask about home adherence and problems encountered in order to increase adherence to frequency per week [86].

Our findings also shed light on the importance of varying the physical activities offered to children with DD, according to an individualized approach, to include playful activities such as dance, aquatic or outdoors, allowing them to develop multiple components of physical literacy [68,70,78,83,84]. Moreover, children with DD would benefit from more time outdoors (or at least in open spaces), smaller group settings and portable equipment (e.g., balls instead of fixed playgrounds) in preschool to enhance their physical activity participation [72]. However, preschool teachers or staff involved in leisure activities may require training and support to incorporate these activities into their daily routine [68]. For example, some training is available to teachers on how to create an empowering motivational climate to support students’ motivation and engagement in physical education [109], and for camp facilitators to develop the inclusive physical literacy of campers with special needs [106]. Training courses specific to the development of physical literacy are also available online (e.g., “Physical Literacy Leader—Levels 1 to 3”).

Our findings also underscore the relevance of including motor programming in early intervention services for children with DD because of the real benefits it offers [62,70]. Targeting the development of gross motor skills is a promising way to increase these children’s overall physical activity levels. Nevertheless, to promote children’s participation in physical activity, developing both gross and fine motor skills continues to be recommended insofar as both are associated with physical activity and sedentary habits later in life [69]. Because motor and executive tasks share common processes (e.g., inhibition, planning, monitoring) [128], cognitively challenging physical activity programs should be implemented to improve both the executive functions and motor development of children with DD as early as possible [55,129]. Additionally, to help these children take part in physical activity, socio-psychological aspects, which are also negatively affected by their disorder, need to be considered [74].

## 5. Conclusions

In conclusion, the unique contribution of this review lies in its integrative overview of physical literacy and physical activity research in early childhood among children with DD. By bringing together and mapping a fragmented and conceptually heterogeneous body of literature, this review provides one of the few comprehensive overviews of how physical literacy has been conceptualized in this population. By focusing specifically on children aged 0–6 years, a critical yet largely underrepresented developmental period, this review clarifies the current state of an emerging field, identifies important conceptual gaps, and establishes a foundational evidence base to inform future research, and early intervention strategies. In this regard, all stakeholders involved in the care of young children with DD (e.g., parents, teachers, childcare educators, etc.) are encouraged to offer varied positive and inclusive physical activity opportunities that respect children’s abilities and individual needs.

## Figures and Tables

**Table 1:** Description of the components of the Canadian consensus framework [43].

Components	Description
Behavioral	Taking personal responsibility for physical literacy by freely choosing to be active on a regular basis (e.g., prioritizing and sustaining involvement in a range of meaningful and personally challenging activities)
Physical	Being able to develop movement skills and patterns, and the capacity to experience a variety of movement intensities and durations
Cognitive	Identifying and expressing the essential qualities that influence movement, understanding the health benefits of an active lifestyle and appreciating appropriate safety features associated with physical activity in a variety of contexts
Affective	Demonstrating enthusiasm, enjoyment and self-assurance in adopting physical activity as an integral part of life

**Table 2:** Inclusion and exclusion criteria.

	Inclusion	Exclusion	Justification
Population	Children with DD six years old and under	Children without DD or over six years old	Population targeted in the study
Years of publication	Articles published between 2010 and 2026	Articles published before 2010	Most up to date scientific knowledge
Type of study	Empirical studies	Non-empirical studies	Evidence-based outcomes
Context	Involving physical activity	Not involving physical activity	Subject targeted in the study
Language	Articles written in English	Other language	The most common scientific language

**Table 3:** Number of articles published per year.

Year	Frequency (*n*)
2010	1
2011	3
2012	1
2013	2
2014	0
2015	3
2016	1
2017	6
2018	1
2019	0
2020	1
2021	2
2022	0
2023	2
2024	1
2025	1
2026	0

## Data Availability

The original contributions presented in this study are included in the article/Supplementary Materials. Further inquiries can be directed to the corresponding author.

## Supplementary Materials

The following supporting information can be downloaded at: https://www.mdpi.com/article/10.3390/children13040548/s1, Table S1: Preferred Reporting Items for Systematic reviews and Meta-Analyses extension for Scoping Reviews (PRISMA-ScR) Checklist; Table S2: Full search strategy; Table S3: References for retained articles; Table S4: Summary of selected studies; Table S5: Programs’ description.

## Abbreviations

The following abbreviations are used in this manuscript:

ASD	Autism spectrum disorder
ADHD	Attention-deficit/hyperactivity disorder
DCD	Developmental coordination disorder
DD	Developmental disabilities
DS	Down syndrome
ID	Intellectual disability
TD	Typical development

## References

[1] Bloemen M.A., Backx F.J., Takken T., Wittink H., Benner J., Mollema J., de Groot J.F. (2015). Factors associated with physical activity in children and adolescents with a physical disability: A systematic review. Dev. Med. Child Neurol..

[2] Rico-Gonzalez M., Holsbrekken E., Martin-Moya R., Ardigo L.P. (2025). Interventions for reducing screen time of preschoolers: A systematic review of randomized controlled trials. J. Prim. Care Community Health.

[3] Ash T., Agaronov A., Young T., Aftosmes-Tobio A., Davison K.K. (2017). Family-based childhood obesity prevention interventions: A systematic review and quantitative content analysis. Int. J. Behav. Nutr. Phys. Act..

[4] ParticipACTION Rallying for Resilience. Keeping Children and Youth Active in a Changing Climate. https://www.participaction.com/wp-content/uploads/2024/05/2024-Children-and-Youth-Report-Card-Technical-Report.pdf.

[5] Chaput J.P., Colley R.C., Aubert S., Carson V., Janssen I., Roberts K.C., Tremblay M.S. (2017). Proportion of preschool-aged children meeting the Canadian 24-Hour Movement Guidelines and associations with adiposity: Results from the Canadian Health Measures Survey. BMC Public Health.

[6] Tapia-Serrano M.A., Sevil-Serrano J., Sanchez-Miguel P.A., Lopez-Gil J.F., Tremblay M.S., Garcia-Hermoso A. (2022). Prevalence of meeting 24-Hour Movement Guidelines from pre-school to adolescence: A systematic review and meta-analysis including 387,437 participants and 23 countries. J. Sport Health Sci..

[7] Tremblay M.S., Carson V., Chaput J.P., Connor Gorber S., Dinh T., Duggan M., Faulkner G., Gray C.E., Gruber R., Janson K. (2016). Canadian 24-Hour movement guidelines for children and youth: An integration of physical activity, sedentary behaviour, and sleep. Appl. Physiol. Nutr. Metab..

[8] Shikako-Thomas K., Majnemer A., Law M., Lach L. (2008). Determinants of participation in leisure activities in children and youth with cerebral palsy: Systematic review. Phys. Occup. Ther. Pediatr..

[9] Colley R.C., Butler G., Garriguet D., Prince S.A., Roberts K.C. (2019). Comparison of self-reported and accelerometer-measured physical activity among Canadian youth. Health Rep..

[10] Arbour-Nicitopoulos K., Grassmann V., Orr K., McPherson A.C., Faulkner G.E., Wright F.V. (2018). A scoping review of inclusive out-of-school time physical activity programs for children and youth with physical disabilities. Adapt. Phys. Act. Q..

[11] Mak T.C.T., Chan D.K.C., Capio C.M., Yoong S.L., Nathan N., Golley R. (2021). Strategies for teachers to promote physical activity in early childhood education settings—A scoping review. Int. J. Environ. Res. Public Health.

[12] Hardie Murphy M., Rowe D.A., Woods C.B. (2016). Sports participation in youth as a predictor of physical activity: A 5-year longitudinal study. J. Phys. Act. Health.

[13] Humphreys B.R., McLeod L., Ruseski J.E. (2014). Physical activity and health outcomes: Evidence from Canada. Health Econ..

[14] Ekblom-Bak E., Ekblom O., Andersson G., Wallin P., Ekblom B. (2018). Physical education and leisure-time physical activity in youth are both important for adulthood activity, physical performance, and health. J. Phys. Act. Health.

[15] Chaput J.-P., Willumsen J., Bull F., Chou R., Ekelund U., Firth J., Jago R., Ortega F.B., Katzmarzyk P.T. (2020). 2020 WHO guidelines on physical activity and sedentary behaviour for children and adolescents aged 5–17 years: Summary of the evidence. Int. J. Behav. Nutr. Phys. Act..

[16] Carson V., Lee E.Y., Hewitt L., Jennings C., Hunter S., Kuzik N., Stearns J.A., Unrau S.P., Poitras V.J., Gray C. (2017). Systematic review of the relationships between physical activity and health indicators in the early years (0–4 years). BMC Public Health.

[17] Taylor R.W., Haszard J.J., Healey D., Meredith-Jones K.A., Taylor B.J., Galland B.C. (2021). Adherence to 24-h movement behavior guidelines and psychosocial functioning in young children: A longitudinal analysis. Int. J. Behav. Nutr. Phys. Act..

[18] Cliff D.P., McNeill J., Vella S.A., Howard S.J., Santos R., Batterham M., Melhuish E., Okely A.D., de Rosnay M. (2017). Adherence to 24-Hour Movement Guidelines for the Early Years and associations with social-cognitive development among Australian preschool children. BMC Public Health.

[19] Christian H., Murray K., Trost S.G., Schipperijn J., Trapp G., Maitland C., Divitini M. (2022). Meeting the australian 24-hour movement guidelines for the early years is associated with better social-emotional development in preschool boys. Prev. Med. Rep..

[20] ParticipACTION Le Rôle de la Famille Dans L’activité Physique, les Comportements Sédentaires et le Sommeil des Enfants et des Jeunes. L’édition 2020 du Bulletin de L’activité Physique Chez les Enfants et les Jeunes de ParticipACTION. https://participaction.cdn.prismic.io/participaction/3b498307-98c1-4210-8155-69322766799f_Bulletin_complet.pdf.

[21] World Health Organization (2019). Guidelines on Physical Activity, Sedentary Behaviour and Sleep for Children Under 5 Years of Age.

[22] Aitchison B., Rushton A.B., Martin P., Barr M., Soundy A., Heneghan N.R. (2022). The experiences and perceived health benefits of individuals with a disability participating in sport: A systematic review and narrative synthesis. Disabil. Health J..

[23] Rico-González M., Goth U.S., Martín-Moya R., Ardigò L.P. (2025). The relationship with meeting physical activity guidelines in preschool-aged children: A systematic review. Pediatr. Rep..

[24] Pica R. (2011). Why preschoolers need physical education. Young Child..

[25] Diaz K.M. (2020). Physical inactivity among parents of children with and without Down syndrome: The National Health Interview Survey. J. Intellect. Disabil. Res..

[26] Cairney J., Clark H., Dudley D., Kriellaars D. (2019). Physical literacy in children and youth—A construct validation study. J. Teach. Phys. Educ..

[27] Sport for Life Physical Literacy—Sport for Life. https://sportforlife.ca/physical-literacy/.

[28] Palmer K.K., Chinn K.M., Robinson L.E. (2019). The effect of the CHAMP intervention on fundamental motor skills and outdoor physical activity in preschoolers. J. Sport Health Sci..

[29] Eggleston M., Watkins W., Frampton C., Hanger N. (2020). Coordination difficulties and self-esteem: The views of children, adolescents, and their parents. Aust. Occup. Ther. J..

[30] Noetel M., Sanders T., Gallardo-Gomez D., Taylor P., Del Pozo Cruz B., van den Hoek D., Smith J.J., Mahoney J., Spathis J., Moresi M. (2024). Effect of exercise for depression: Systematic review and network meta-analysis of randomised controlled trials. BMJ.

[31] Ku B., MacDonald M., Hatfield B., Gunter K. (2020). Parental influence on the physical activity behaviors of young children with developmental disabilities. Adapt. Phys. Act. Q..

[32] American Psychological Association APA Dictionary of Psychology. https://dictionary.apa.org/developmental-disability.

[33] Bandini L.G., Gleason J., Curtin C., Lividini K., Anderson S.E., Cermak S.A., Maslin M., Must A. (2013). Comparison of physical activity between children with autism spectrum disorders and typically developing children. Autism.

[34] Missiuna C., Cairney J., Pollock N., Campbell W., Russell D.J., Macdonald K., Schmidt L., Heath N., Veldhuizen S., Cousins M. (2014). Psychological distress in children with developmental coordination disorder and attention-deficit hyperactivity disorder. Res. Dev. Disabil..

[35] Cavalcante-Neto J.L., Bourke M., Silva J.M.C., Cairney J. (2026). Emotional outcomes are poorer in adults with developmental coordination disorder: A systematic review and meta-analyses. J. Psychosom. Res..

[36] Esentürk O.K. (2021). Parents’ perceptions on physical activity for their children with autism spectrum disorders during the novel Coronavirus outbreak. Int. J. Dev. Disabil..

[37] Edwards L.C., Bryant A.S., Keegan R.J., Morgan K., Cooper S.M., Jones A.M. (2018). Measuring physical literacy and related constructs: A systematic review of empirical findings. Sports Med..

[38] Whitehead M. (2013). Definition of physical literacy and clarification of related issues. J. Sport Sci. Phys. Educ..

[39] Britton Ú., Onibonoje O., Belton S., Behan S., Peers C., Issartel J., Roantree M. (2023). Moving well-being well: Using machine learning to explore the relationship between physical literacy and well-being in children. Appl. Psychol. Health Wellbeing.

[40] Sport for Life Developing Physical Literacy: Building a New Normal for All Canadians. https://sportforlife.ca/wp-content/uploads/2019/09/DPL-2_2021.pdf.

[41] International Physical Literacy Association http://www.physical-literacy.org.uk.

[42] Sport for Life PLAY Stands for Physical Literacy Assessment for Youth. https://play.physicalliteracy.ca/.

[43] Sport for Life Society, Physical Literacy for Life What Is Physical Literacy?. https://physicalliteracy.ca/physical-literacy/.

[44] Whitehead M. (2013). Stages in physical literacy journey. J. Sport Sci. Phys. Educ..

[45] Goodway J.D., Brian A., Chang S.H., Famelia R., Tsuda E., Robinson L.E. (2013). Promoting physical literacy in the early years through project SKIP. J. Sport Sci. Phys. Educ..

[46] Edwards L.C., Bryant A.S., Keegan R.J., Morgan K., Jones A.M. (2017). Definitions, foundations and associations of physical literacy: A systematic review. Sports Med..

[47] Cairney J., Dudley D., Kwan M., Bulten R., Kriellaars D. (2019). Physical literacy, physical activity and health: Toward an evidence-informed conceptual model. Sports Med..

[48] López-Gajardo M.Á., Tapia-Serrano M.Á., Burgueño R., Abós Á., Leo F.M., Sánchez-Miguel P.A., García-González L. (2025). Understanding motivational dynamics: Longitudinal associations between motivation and physical activity in children. Psychol. Sport Exerc..

[49] Fortnum K., Furzer B., Reid S., Jackson B., Elliott C. (2018). The physical literacy of children with behavioural and emotional mental health disorders: A scoping review. Ment. Health Phys. Act..

[50] Mazzoli E., Contardo Ayala A.M., Koorts H., Timperio A., Lander N., Lubans D.R., Ridgers N.D., Anderson K.L.M., Cairney J., Barnett L.M. (2025). School-based strategies to increase physical activity and reduce sedentary behaviour in students with disability: Protocol of theTransformUs All Abilities hybrid type II implementation-effectiveness trial. BMJ Open.

[51] Bopp T., Stellefson M., Weatherall B., Spratt S. (2019). Promoting physical literacy for disadvantaged youth living with chronic disease. Am. J. Health Educ..

[52] Fortnum K., Weber M.D., Dudley D., Tudella E., Kwan M., Richard V., Cairney J. (2025). Physical literacy, Physical activity, and health: A citation content analysis and narrative review. Sports Med.—Open.

[53] Carl J., Barratt J., Wanner P., Topfer C., Cairney J., Pfeifer K. (2022). The effectiveness of physical literacy interventions: A systematic review with meta-analysis. Sports Med..

[54] Li J., Potter M., Hwang Y., Boyd M., Moldenhauer R., Hills J., Naylor P.J., Rhodes R.E., Liu S., Buckler J. (2025). Individual and environmental correlates of physical literacy sub-components in early childhood. J. Sports Sci..

[55] Chen H., Liang Q., Wang B., Liu H., Dong G., Li K. (2024). Sports game intervention aids executive function enhancement in children with autism—An fNIRS study. Neurosci. Lett..

[56] Zhang W., Cai K., Xiong X., Zhu L., Sun Z., Yang S., Cheng W., Mao H., Chen A. (2025). Alterations of triple network dynamic connectivity and repetitive behaviors after mini-basketball training program in children with autism spectrum disorder. Sci. Rep..

[57] Peters M.D., Godfrey C.M., Khalil H., McInerney P., Parker D., Soares C.B. (2015). Guidance for conducting systematic scoping reviews. Int. J. Evid. Based Healthc..

[58] Arksey H., O’Malley L. (2005). Scoping studies: Towards a methodological framework. Int. J. Soc. Res. Methodol..

[59] Munn Z., Peters M.D.J., Stern C., Tufanaru C., McArthur A., Aromataris E. (2018). Systematic review or scoping review? Guidance for authors when choosing between a systematic or scoping review approach. BMC Med. Res. Methodol..

[60] Tricco A.C., Lillie E., Zarin W., O’Brien K.K., Colquhoun H., Levac D., Moher D., Peters M.D.J., Horsley T., Weeks L. (2018). PRISMA extension for scoping reviews (PRISMA-ScR): Checklist and explanation. Ann. Intern. Med..

[61] Braun V., Clarke V. (2006). Using thematic analysis in psychology. Qual. Res. Psychol..

[62] Favazza P.C., Siperstein G.N. (2013). Young athletes: A special olympics motor skill development program. State Educ. Stand..

[63] Jarus T., Lourie-Gelberg Y., Engel-Yeger B., Bart O. (2011). Participation patterns of school-aged children with and without DCD. Res. Dev. Disabil..

[64] Lin C.Y., Chang Y.M. (2015). Interactive augmented reality using Scratch 2.0 to improve physical activities for children with developmental disabilities. Res. Dev. Disabil..

[65] Young A., Healy S., Silliman-French L., Brian A. (2021). A pilot study of a parent-mediated, web-based motor skill intervention for children with down syndrome: Project SKIP. Adapt. Phys. Act. Q..

[66] Hastie P.A., Rudisill M.E., Boyd K. (2016). An ecological analysis of a preschool mastery climate physical education programme. Phys. Educ. Sport Pedagog..

[67] Becerra L.A., Higbee T.S., Vieira M.C., Pellegrino A.J., Hobson K. (2021). The effect of photographic activity schedules on moderate-to-vigorous physical activity in children with autism spectrum disorder. J. Appl. Behav. Anal..

[68] Nelson C., Paul K., Johnston S.S., Kidder J.E. (2017). Use of a creative dance intervention package to increase social engagement and play complexity of young children with autism spectrum disorder. Educ. Train. Autism Dev. Disabl..

[69] Sánchez G.F.L., Williams G., Aggio D., Vicinanza D., Stubbs B., Kerr C., Johnstone J., Roberts J., Smith L. (2017). Prospective associations between measures of gross and fine motor coordination in infants and objectively measured physical activity and sedentary behavior in childhood. Medicine.

[70] Ketcheson L., Hauck J., Ulrich D. (2017). The effects of an early motor skill intervention on motor skills, levels of physical activity, and socialization in young children with autism spectrum disorder: A pilot study. Autism.

[71] Ketcheson L., Hauck J.L., Ulrich D. (2018). The levels of physical activity and motor skills in young children with and without autism spectrum disorder, aged 2–5 years. Autism.

[72] Schenkelberg M.A., McIver K.L., Brown W.H., Pate R.R. (2020). Preschool environmental influences on physical activity in children with disabilities. Med. Sci. Sports Exerc..

[73] Ketcheson L., Staples K., Pitchford E.A., Loetzner F. (2023). Promoting positive health outcomes in an urban community-based physical activity intervention for preschool aged children on the autism spectrum. J. Autism Dev. Disord..

[74] Liberman L., Ratzon N., Bart O. (2013). The profile of performance skills and emotional factors in the context of participation among young children with Developmental Coordination Disorder. Res. Dev. Disabil..

[75] Kambas A., Venetsanou F., Kelaraki D., Karageorgopoulou M. (2025). Group psychomotor therapy improves socio-emotional and motor competence of pre-school aged children, with and without attention deficit hyperactivity disorder. Body Mov. Dance Psychother..

[76] Draudvilienė L., Draudvila J., Stankevičiūtė S., Daniusevičiūtė-Brazaitė L. (2024). Two physiotherapy methods to improve the physical condition of children with autism spectrum disorder. Children.

[77] Salem Y., Gropack S.J., Coffin D., Godwin E.M. (2012). Effectiveness of a low-cost virtual reality system for children with developmental delay: A preliminary randomised single-blind controlled trial. Physiotherapy.

[78] Takahashi H., An M., Matsumura T., Seki M., Ogawa Y., Sasai T., Matsushima K., Tabata A., Kato T. (2023). Effectiveness of dance/movement therapy intervention for children with intellectual disability at an early childhood special education preschool. Am. J. Danc. Ther..

[79] Karanth P., Shaista S., Srikanth N. (2010). Efficacy of communication DEALL—An indigenous early intervention program for children with autism spectrum disorders. Indian J. Pediatr..

[80] Bart O., Jarus T., Erez Y., Rosenberg L. (2011). How do young children with DCD participate and enjoy daily activities?. Res. Dev. Disabil..

[81] Lakes K.D., Abdullah M.M., Youssef J., Donnelly J.H., Taylor-Lucas C., Goldberg W.A., Cooper D., Radom-Aizik S. (2017). Assessing parent perceptions of physical activity in families of toddlers with neurodevelopmental disorders: The Parent Perceptions of Physical Activity Scale (PPPAS). Pediatr. Exerc. Sci..

[82] Kennedy-Behr A., Rodger S., Mickan S. (2015). Play or hard work: Unpacking well-being at preschool. Res. Dev. Disabil..

[83] Fabrizi S.E. (2015). Splashing our way to playfulness! An aquatic playgroup for young children with autism, a repeated measures design. J. Occup. Ther. Sch. Early Interv..

[84] Zachor D.A., Vardi S., Baron-Eitan S., Brodai-Meir I., Ginossar N., Ben-Itzchak E. (2017). The effectiveness of an outdoor adventure programme for young children with autism spectrum disorder: A controlled study. Dev. Med. Child Neurol..

[85] LaVesser P., Berg C. (2011). Participation patterns in preschool children with an autism spectrum disorder. OTJR Occup. Particip. Health.

[86] Medina-Mirapeix F., Lillo-Navarro C., Montilla-Herrador J., Gacto-Sánchez M., Franco-Sierra M.Á., Escolar-Reina P. (2017). Predictors of parents’ adherence to home exercise programs for children with developmental disabilities, regarding both exercise frequency and duration: A survey design. Eur. J. Phys. Rehabil. Med..

[87] An M., Tanaka R., Hirota N., Sasai T., Takahashi H., Ogawa Y., Horai S., Inoue M., Rakwal R., Kato T. (2024). A scoping review of adapted physical activity interventions for children and youth with disabilities using international classification of functioning, disability and health: Children and youth version as a reference. Int. J. Dev. Disabil..

[88] D’Amours J., Girard S., Miquelon P., Veillette P.-L. (2025). Effects of group-based physical activity programs on children, adolescents, and young adults with disabilities: A systematic review. PLoS ONE.

[89] Ge W., Zhang C., Yang G., Zhang B. (2024). Prevalence and trends of autism spectrum disorder and other developmental disabilities among children and adolescents in the United States from 2019 to 2021. Front. Psychiatry.

[90] World Health Organization Autism Spectrum Disorders. https://www.who.int/news-room/fact-sheets/detail/autism-spectrum-disorders.

[91] D’Amours J., Girard S., Miquelon P., Veillette P.-L. (2025). Bonheur en boule: An adapted group-based physical activity program for youth with disabilities. Front. Sports Act. Living.

[92] Cairney J., Clark H.J., James M.E., Mitchell D., Dudley D.A., Kriellaars D. (2018). The preschool physical literacy assessment tool: Testing a new physical literacy tool for the early years. Front. Pediatr..

[93] Weerackody S.C., Clutterbuck G.L., Johnston L.M. (2023). Measuring psychological, cognitive, and social domains of physical literacy in school-aged children with neurodevelopmental disabilities: A systematic review and decision tree. Disabil. Rehabil..

[94] Saxena S., Shikako Thomas K. (2020). Physical literacy programs for children with disabilities: A realist review. Leisure/Loisir.

[95] Ministère de l’Éducation Programme-Cycle de L’éducation Préscolaire [Preschool Education Program-Cycle]. https://cdn-contenu.quebec.ca/cdn-contenu/education/pfeq/prescolaire/Programme-cycle-prescolaire.pdf.

[96] Ministère de la Famille du Québec Accueillir la Petite Enfance: Programme Éducatif Pour les Services de Garde du Québec [Welcoming the Early Years: Quebec’s Child Care Education Program]. https://cdn-contenu.quebec.ca/cdn-contenu/adm/min/famille/publications-adm/Service_de_garde/programme_educatif.pdf.

[97] Kowalski A.J., Armstrong B., Trude A.C.B., Arbaiza R., Czinn A., Bellows L.L., Johnson S.L., Wang Y., Hager E.R., Black M.M. (2025). Creating healthy habits for Maryland preschoolers (CHAMP): A cluster-randomized controlled trial among childcare centers. Int. J. Behav. Nutr. Phys. Act..

[98] Biddle S.J.H., Gorely T., Papaioannou A., Hackfort D. (2014). Sitting psychology: Towards a psychology of sedentary behaviour. Routledge Companion to Sport and Exercise Psychology: Global Perspectives and Fundamental Concepts.

[99] Mota J.G., Tassitano R.M., Lemos L., Okely A.D., Coppens E., Santos E.A., Mota J., Lenoir M., Lucena Martins C.M. (2025). Compliance with the 24-h movement behaviours guidelines among brazilian toddlers. Child Care Health Dev..

[100] Tucker P., Vanderloo L.M., Johnson A.M., Burke S.M., Irwin J.D., Gaston A., Driediger M., Timmons B.W. (2017). Impact of the Supporting Physical Activity in the Childcare Environment (SPACE) intervention on preschoolers’ physical activity levels and sedentary time: A single-blind cluster randomized controlled trial. Int. J. Behav. Nutr. Phys. Act..

[101] Girard S., Paquet A., Cyr C. (2022). Camps de jour et enfants ayant un trouble du spectre de l’autisme: Examen de la portée de la littérature scientifique. Leisure/Loisir.

[102] Alonso-Martinez L., Ramirez-Velez R., Garcia-Hermoso A., Izquierdo M., Alonso-Martinez A.M. (2025). Improving preschool fundamental motor skills through interventions targeting 24-hour movement behaviors and socioecological factors: A meta-analysis. J. Phys. Act. Health.

[103] Grauduszus M., Wessely S., Klaudius M., Joisten C. (2023). Definitions and assessments of physical literacy among children and youth: A scoping review. BMC Public Health.

[104] Stuckey M., Richard V., Decker A., Aubertin P., Kriellaars D. (2021). Supporting holistic wellbeing for performing artists during the COVID-19 pandemic and recovery: Study protocol. Front. Psychol..

[105] Jefferies P., Ungar M., Aubertin P., Kriellaars D. (2019). Physical literacy and resilience in children and youth. Front. Public Health.

[106] Girard S., Paquet A., McKinnon S., Rousseau M. (2023). Supporting inclusive physical literacy development in leisure settings: Building on the affective dimension. PHEnex J..

[107] Martin J.J., Choi Y.S. (2009). Parents’ physical activity−related perceptions of their children with disabilities. Disabil. Health J..

[108] Teraoka E., Kirk D. (2022). Exploring pupils’ and physical education teachers’ views on the contribution of physical education to Health and Wellbeing in the affective domain. Sport Educ. Soc..

[109] Girard S., Desbiens J.-F., Hogue A.-M. (2023). Effects of a training course on creation of an empowering motivational climate in physical education: A quasi-experimental study. Phys. Educ. Sport Pedagog..

[110] Ames C. (1992). Classrooms: Goals, structures, and student motivation. J. Educ. Psychol..

[111] Bolter N.D., Kipp L.E. (2016). Sportspersonship coaching behaviours, relatedness need satisfaction, and early adolescent athletes’ prosocial and antisocial behaviour. Int. J. Sport Exerc. Psychol..

[112] Sparks C., Lonsdale C., Dimmock J., Jackson B. (2017). An intervention to improve teachers’ interpersonally involving instructional practices in high school physical education: Implications for student relatedness support and in-class experiences. J. Sport Exerc. Psychol..

[113] Schenkelberg M.A., Rosenkranz R.R., Milliken G.A., Dzewaltowski D.A. (2015). Social environmental influences on physical activity of children with autism spectrum disorders. J. Phys. Act. Health.

[114] Schlechter C.R., Rosenkranz R.R., Fees B.S., Dzewaltowski D.A. (2017). Preschool daily patterns of physical activity driven by location and social context. J. Sch. Health.

[115] Diamond K.E., Hong S.-Y. (2010). Young children’s decisions to include peers with physical disabilities in play. J. Early Interv..

[116] Stoloff D., Stoloff S. (2023). Le Langage du Corps: Vivre des Histoires en Mouvement Avec les Enfants: 2–6 Ans.

[117] Larin M., Stoloff S., Girard S. (2025). Quand la magie d’une histoire se vit dans le gymnase…. Propulsion.

[118] Eisenburger N., Jáuregui Ulloa E., Villegas Balderrama C.V., Villegas Balderrama K.J., Muñoz Rodríguez S.N., Calderón Escalante A., López Alonso S.J., Orona Escápite A., Flores Olivares L.A., Muñoz De la Riva M. (2024). Addressing physical inactivity in Mexican children: The role of parents and their physical literacy. Obes. Sci. Pract..

[119] Ku B., Rhodes R.E. (2020). Physical activity behaviors in parents of children with disabilities: A systematic review. Res. Dev. Disabil..

[120] Rhodes R.E., Guerrero M.D., Vanderloo L.M., Barbeau K., Birken C.S., Chaput J.-P., Faulkner G., Janssen I., Madigan S., Mâsse L.C. (2020). Development of a consensus statement on the role of the family in the physical activity, sedentary, and sleep behaviours of children and youth. Int. J. Behav. Nutr. Phys. Act..

[121] Su D.L.Y., Tang T.C.W., Chung J.S.K., Lee A.S.Y., Capio C.M., Chan D.K.C. (2022). Parental influence on child and adolescent physical activity level: A meta-analysis. Int. J. Environ. Res. Public Health.

[122] Block M.E., Taliaferro A., Moran T. (2013). Physical activity and youth with disabilities: Barriers and supports. Prev. Res..

[123] MacEachern S., Forkert N.D., Lemay J.-F., Dewey D. (2022). Physical activity participation and barriers for children and adolescents with disabilities. Intl. J. Disabil. Dev. Educ..

[124] Rimmer J.A., Rowland J.L. (2008). Physical activity for youth with disabilities: A critical need in an underserved population. Dev. Neurorehabil..

[125] Rimmer J.H., Marques A.C. (2012). Physical activity for people with disabilities. Lancet.

[126] Rimmer J.H., Padalabalanarayanan S., Malone L.A., Mehta T. (2017). Fitness facilities still lack accessibility for people with disabilities. Disabil. Health J..

[127] Ministère de la Santé et des Services Sociaux Stratégie Nationale de Prévention en Santé 2025–2035. https://publications.msss.gouv.qc.ca/msss/fichiers/2025/25-297-04W.pdf.

[128] van der Fels I.M.J., te Wierike S.C.M., Hartman E., Elferink-Gemser M.T., Smith J., Visscher C. (2015). The relationship between motor skills and cognitive skills in 4–16 year old typically developing children: A systematic review. J. Sci. Med. Sport.

[129] Alesi M., Pecoraro D., Pepi A. (2019). Executive functions in kindergarten children at risk for developmental coordination disorder. Eur. J. Spec. Needs Educ..

