# Supplementary material for: Physical Literacy and Physical Activity of Young Children with Developmental Disabilities: A Scoping Review
Source: Children (Basel). 2026 Apr 15;13(4):548. doi: 10.3390/children13040548 (PMC13115258; doi:10.3390/children13040548)
Supplement: Supplementary file 1 [file children-13-00548-s001.zip › Supplementary Table S2.pdf]

Supplementary Table S2. Full search strategy

****An updated search was conducted in each database in 2024 and 2026 using the same search strategies as the initial search to ensure the review remained up to date.*

# S1) inclusion & developmental disabilities, S2) physical literacy, S3) physical activity, S4) children.

Search dates for all databases including updates: 13 July 2020 / March 2024 and January 2026

PsycINFO –

#	Search	Results	Database	Notes
S1	TI (Inclusion OR "Special need*" OR Autism* OR "Developmental disorder*" OR "Intellectual disability" OR Adapted OR "Developmental disabilit*" OR "Developmental delay" OR "Autism Spectrum Disorders" OR "Adaptive Behavior Measures" OR "Intellectual development disorder" OR "Delayed development") OR SU (Inclusion OR "Special need*" OR Autism* OR "Developmental disorder*" OR "Intellectual disability" OR Adapted OR "Developmental disabilit*" OR "Developmental delay" OR "Autism Spectrum Disorders" OR "Adaptive Behavior Measures" OR "Intellectual development disorder" OR "Delayed development") OR AB (Inclusion OR "Special need*" OR Autism* OR "Developmental disorder*" OR "Intellectual disability" OR Adapted OR "Developmental disabilit*" OR "Developmental delay" OR "Autism Spectrum Disorders"	204,170	APA PsycInfo	

#	Search	Results	Database	Notes
	OR "Adaptive Behavior Measures" OR "Intellectual development disorder" OR "Delayed development")			
S2	TI ("physical literacy" OR Adequacy OR confidence OR enjoyment OR motivation OR "perceived competence" OR "self-efficacy" OR predilection OR knowledge OR understanding OR "self-perception" OR "self-concept" OR "self-esteem* " OR Affectiv* OR Cognitive) OR SU ("physical literacy" OR Adequacy OR confidence OR enjoyment OR motivation OR "perceived competence" OR "self-efficacy" OR predilection OR knowledge OR understanding OR "self-perception" OR "self-concept" OR "self-esteem* " OR Affectiv* OR Cognitive) OR AB ("physical literacy" OR Adequacy OR confidence OR enjoyment OR motivation OR "perceived competence" OR "self-efficacy" OR predilection OR knowledge OR understanding OR "self-perception" OR "self-concept" OR "self-esteem* " OR Affectiv* OR Cognitive)	1,439,988	APA PsycInfo	
S3	TI (" Physical activit* " OR "Active play" OR exercise OR Recreation OR "Recreational activit*" OR "day camp" OR "summer camp" OR leisure OR "physical activity experiences") OR SU (" Physical activit* " OR "Active play" OR exercise OR Recreation OR "Recreational activit*" OR "day camp" OR "summer camp" OR leisure OR "physical activity experiences") OR AB (" Physical activit* " OR "Active play" OR exercise OR Recreation OR "Recreational activit*" OR "day camp" OR "summer camp" OR leisure OR "physical activity experiences")	117,804	APA PsycInfo	

Medline –

DE and MeSH

#	Search	Results	Database	Notes
S1	TI (Inclusion OR "Special need*" OR Autism* OR "Autistic Disorder" OR "Developmental disorder*" OR "Intellectual disability" OR Adapted OR "Pervasive Developmental Disorders" OR "Developmental disabilit*" OR "Developmental delay" OR "Autism Spectrum Disorders" OR "Autism Spectrum Disorder" OR "Adaptive Behavior Measures" OR "Intellectual development disorder" OR "Delayed development") OR SU (Inclusion OR "Special need*" OR Autism* OR "Autistic Disorder" OR "Developmental disorder*" OR "Intellectual disability" OR Adapted OR "Pervasive Developmental Disorders" OR "Developmental disabilit*" OR "Developmental delay" OR "Autism Spectrum Disorders" OR "Autism Spectrum Disorder" OR "Adaptive Behavior Measures" OR "Intellectual development disorder" OR "Delayed development") OR AB (Inclusion OR "Special need*" OR Autism* OR "Autistic Disorder" OR "Developmental disorder*" OR "Intellectual disability" OR Adapted OR "Pervasive Developmental Disorders" OR	(52,138)	MEDLINE	

#	Search	Results	Database	Notes
	"Developmental disabilit*" OR "Developmental delay" OR "Autism Spectrum Disorders" OR "Autism Spectrum Disorder" OR "Adaptive Behavior Measures" OR "Intellectual development disorder" OR "Delayed development")			
S2	TI ("physical literacy" OR Adequacy OR confidence OR enjoyment OR "Pleasure" OR motivation OR "perceived competence" OR "Self-Efficacy" OR predilection OR knowledge OR understanding OR "Comprehension" OR "self-perception" OR "Self Concept" OR "self-esteem* " OR Affectiv* OR "Affective Behavior" OR Cognitive OR "Cognitive Ability") OR SU ("physical literacy" OR Adequacy OR confidence OR enjoyment OR "Pleasure" OR motivation OR "perceived competence" OR "Self-Efficacy" OR predilection OR knowledge OR understanding OR "Comprehension" OR "self-perception" OR "Self Concept" OR "self-esteem* " OR Affectiv* OR "Affective Behavior" OR Cognitive OR "Cognitive Ability") OR AB ("physical literacy" OR Adequacy OR confidence OR enjoyment OR "Pleasure" OR motivation OR "perceived competence" OR "Self-Efficacy" OR predilection OR knowledge OR understanding OR "Comprehension" OR "self-perception" OR "Self Concept" OR "self-esteem* " OR Affectiv* OR "Affective Behavior" OR Cognitive OR "Cognitive Ability")	(136,124)		

#	Search	Results	Database	Notes
S3	TI ("Physical activit* " OR "Exercise" OR "Active play" OR Recreation OR "Recreational activit*" OR "day camp" OR "Program Development" OR "summer camp" OR "Summer Programs" OR leisure OR "Leisure Activities") OR SU ("Physical activit* " OR "Exercise" OR "Active play" OR Recreation OR "Recreational activit*" OR "day camp" OR "Program Development" OR "summer camp" OR "Summer Programs" OR leisure OR "Leisure Activities") OR AB ("Physical activit* " OR "Exercise" OR "Active play" OR Recreation OR "Recreational activit*" OR "day camp" OR "Program Development" OR "summer camp" OR "Summer Programs" OR leisure OR "Leisure Activities")	(23,370)		
S4	TI Child* OR SU Child* OR AB Child*			
S5	S1 AND S2 AND S3 AND S4	527		**Limiters -** Publication Year: 2010-2020; 2026 **Age Related:** All Infant: birth-23 months, Child, Preschool: 2-5 years, Child: 6-12 years

The rerun of the exact search strategy combines articles 2020 to 2024 and 2026 added – 470 articles for a total of 997.

ERIC –

DE in red

# 2	Search	Results	Database	Notes
S1	TI (Inclusion OR "Special need*" OR "Individual Needs" OR "Special Programs" OR "Services" OR Autism* OR "Developmental disorder*" OR "Intellectual disability" OR Adapted OR "Pervasive Developmental Disorders" OR "Developmental disabilit*" OR "Developmental delay" OR "Autism Spectrum Disorders" OR "Adaptive Behavior Measures" OR "Intellectual development disorder" OR "Delayed development") OR SU (Inclusion OR "Special need*" OR "Individual Needs" OR "Special Programs" OR "Services" OR Autism* OR "Developmental disorder*" OR "Intellectual disability" OR Adapted OR "Pervasive Developmental Disorders" OR "Developmental disabilit*" OR "Developmental delay" OR "Autism Spectrum Disorders" OR "Adaptive Behavior Measures" OR "Intellectual development disorder" OR "Delayed development") OR AB (Inclusion OR "Special need*" OR "Individual Needs" OR "Special Programs" OR "Services" OR Autism* OR "Developmental disorder*" OR "Intellectual disability" OR Adapted OR "Pervasive Developmental Disorders" OR "Developmental disabilit*" OR "Developmental delay" OR "Autism Spectrum		ÉRIC<br>213,862	

# 2	Search	Results	Database	Notes
	Disorders" OR "Adaptive Behavior Measures" OR "Intellectual development disorder" OR "Delayed development")			
S2	TI ("physical literacy" OR Adequacy OR confidence OR enjoyment OR motivation OR "perceived competence" OR "Perception" OR "self-efficacy" OR predilection OR knowledge OR understanding OR "Learning" OR "self-perception" OR "self-concept" OR "self-esteem* " OR "Beliefs" OR Affectiv* OR "Affective Behavior" OR Cognitive OR "Cognitive Ability") OR SU ("physical literacy" OR Adequacy OR confidence OR enjoyment OR motivation OR "perceived competence" OR "Perception" OR "self-efficacy" OR predilection OR knowledge OR understanding OR "Learning" OR "self-perception" OR "self-concept" OR "self-esteem* " OR "Beliefs" OR Affectiv* OR "Affective Behavior" OR Cognitive OR "Cognitive Ability") OR AB ("physical literacy" OR Adequacy OR confidence OR enjoyment OR motivation OR "perceived competence" OR "Perception" OR "self-efficacy" OR predilection OR knowledge OR understanding OR "Learning" OR "self-perception" OR "self-concept" OR "self-esteem* " OR "Beliefs" OR Affectiv* OR "Affective Behavior" OR Cognitive OR "Cognitive Ability")		ÉRIC<br>782,253	
S3	TI (" Physical activit* " OR "Physical Activity Level" OR "Active play" OR "Activit*" OR exercise OR		ÉRIC<br>277,216	

# 2	Search	Results	Database	Notes
	Recreation OR “Recreational activit*” OR "Physical recreation" OR "Physical Recreation Programs" OR "day camp" OR"Day Camp Programs" OR "summer camp" OR "Summer Programs" leisure OR "Leisure Time" OR “physical activity experiences”) OR SU(" Physical activit* " OR "Physical Activity Level" OR “Active play” OR "Activit*" OR exercise OR Recreation OR “Recreational activit*” OR "Physical recreation" OR "Physical Recreation Programs" OR "day camp" OR"Day Camp Programs" OR "summer camp" OR "Summer Programs" leisure OR "Leisure Time" OR “physical activity experiences”) OR AB (" Physical activit* " OR "Physical Activity Level" OR “Active play” OR "Activit*" OR exercise OR Recreation OR “Recreational activit*” OR "Physical recreation" OR "Physical Recreation Programs" OR "day camp" OR"Day Camp Programs" OR "summer camp" OR "Summer Programs" leisure OR "Leisure Time" OR “physical activity experiences”)			
S4	TI Child* OR SU Child* OR AB Child*		348,808	47,003
S5	S1 AND S2 AND S3 AND S4		281	**Limiters** - Publication Year: 2010-2020; 2026

The rerun of the exact search strategy combines articles 2020 to 2024 and 2026 added – 233 articles for a total of 514.

CINHAL –

DE in red

#	Search	Results	Database	Notes
S1	TI (Inclusion OR "Social Inclusion" OR "Special need*" OR "Disabled" OR Autism* OR "Autistic Disorder" OR "Developmental disorder*" OR "Child Development Disorders" OR "Intellectual disability" OR Adapted OR "Pervasive Developmental Disorders" OR "Developmental disabilit*" OR "Developmental delay" OR "Autism Spectrum Disorders" OR "Adaptive Behavior Measures" OR "Intellectual development disorder" OR "Delayed development") OR SU (Inclusion OR "Social Inclusion" OR "Special need*" OR "Disabled" OR Autism* OR "Autistic Disorder" OR "Developmental disorder*" OR "Child Development Disorders" OR "Intellectual disability" OR Adapted OR "Pervasive Developmental Disorders" OR "Developmental disabilit*" OR "Developmental delay" OR "Autism Spectrum Disorders" OR "Adaptive Behavior Measures" OR "Intellectual development disorder" OR "Delayed development") OR AB (Inclusion OR "Social Inclusion" OR "Special need*" OR "Disabled" OR Autism* OR "Autistic Disorder" OR "Developmental disorder*" OR "Child Development Disorders" OR "Intellectual disability" OR Adapted OR "Pervasive Developmental Disorders" OR	161,030	CINAHL	

#	Search	Results	Database	Notes
	"Developmental disabilit*" OR "Developmental delay" OR "Autism Spectrum Disorders" OR "Adaptive Behavior Measures" OR "Intellectual development disorder" OR "Delayed development")			
S2	TI ("physical literacy" OR Adequacy OR confidence OR enjoyment OR motivation OR "perceived competence" OR "self-efficacy" OR predilection OR knowledge OR understanding OR "self-perception" OR "Self Concept" OR "self-esteem* " OR Affectiv* OR "Affective Behavior" OR Cognitive OR "Cognitive Ability" OR "Cognition") OR SU (physical literacy" OR Adequacy OR confidence OR enjoyment OR motivation OR "perceived competence" OR "self-efficacy" OR predilection OR knowledge OR understanding OR "self-perception" OR "Self Concept" OR "self-esteem* " OR Affectiv* OR "Affective Behavior" OR Cognitive OR "Cognitive Ability" OR "Cognition") OR AB (physical literacy" OR Adequacy OR confidence OR enjoyment OR motivation OR "perceived competence" OR "self-efficacy" OR predilection OR knowledge OR understanding OR "self-perception" OR "Self Concept" OR "self-esteem* " OR Affectiv* OR "Affective Behavior" OR Cognitive OR "Cognitive Ability" OR "Cognition")	375,272		
S3	TI ("Physical activit* " OR "Physical education" OR "Active play" OR exercise OR Recreation OR "Recreational activit*" OR "day camp" OR "Camps" OR	184,096		

#	Search	Results	Database	Notes
	"Program Development" OR "summer camp" OR "Summer Programs" OR leisure OR "Leisure Activities") OR SU ("Physical activit*" OR "Physical education" OR "Active play" OR exercise OR Recreation OR "Recreational activit*" OR "day camp" OR "Camps" OR "Program Development" OR "summer camp" OR "Summer Programs" OR leisure OR "Leisure Activities") OR AB ("Physical activit* " OR "Physical education" OR "Active play" OR exercise OR Recreation OR "Recreational activit*" OR "day camp" OR "Camps" OR "Program Development" OR "summer camp" OR "Summer Programs" OR leisure OR "Leisure Activities")			
S4	TI Child* OR SU Child* OR AB Child*		512,322	
S5	S1 AND S2 AND S3 AND S4		468	**Limiters** - Publication Year: 2010-2020; 2026

The rerun of the exact search strategy combines articles 2020 to 2024 and 2026 added – 303 articles for a total of 771.
