# Supplementary material for: Physical Literacy and Physical Activity of Young Children with Developmental Disabilities: A Scoping Review
Source: Children (Basel). 2026 Apr 15;13(4):548. doi: 10.3390/children13040548 (PMC13115258; doi:10.3390/children13040548)
Supplement: Supplementary file 1 [file children-13-00548-s001.zip › Supplementary Table S3.pdf]

Supplementary Table S3. *Table of references for retained articles in the scoping*

	References for retained articles
1	Bart, O., Jarus, T., Erez, Y., & Rosenberg, L. (2011). How do young children with DCD participate and enjoy daily activities? *Research in Developmental Disabilities: A Multidisciplinary Journal, 32*(4), 1317-1322. https://dx.doi.org/10.1016/j.ridd.2011.01.039
2	Becerra, L. A., Higbee, T. S., Vieira, M. C., Pellegrino, A. J., & Hobson, K. (2021). The effect of photographic activity schedules on moderate-to-vigorous physical activity in children with autism spectrum disorder. *Journal of Applied Behavior Analysis*, *54*(2), 744-759. https://doi.org/10.1002/jaba.796
3	Draudvilienė, L., Draudvila, J., Stankevičiūtė, S., & Daniusevičiūtė-Brazaitė, L. (2024). Two physiotherapy methods to improve the physical condition of children with autism spectrum disorder. *Children*, *11*(7), 798. https://doi.org/10.3390/children11070798
4	Fabrizi, S. E. (2015). Splashing our way to playfulness! An aquatic playgroup for young children with autism, a repeated measures design. *Journal of Occupational Therapy, Schools & Early Intervention*, *8*(4), 292-306. https://doi.org/10.1080/19411243.2015.1116963
5	Favazza, P. C., & Siperstein, G. N. (2013). Young athletes: A special olympics motor skill development program. *State Education Standard, 13*(1), 28-33. http://www.nasbe.org/our-resources/publications/the-state-education-standard/
6	Hastie, P. A., Rudisill, M. E., & Boyd, K. (2016). An ecological analysis of a preschool mastery climate physical education programme. *Physical Education and Sport Pedagogy, 21*(2), 217-232. https://doi.org/10.1080/17408989.2015.1017454
7	Jarus, T., Lourie-Gelberg, Y., Engel-Yeger, B., & Bart, O. (2011). Participation patterns of school-aged children with and without DCD. *Research in Developmental Disabilities: A Multidisciplinary Journal, 32*(4), 1323-1331. https://doi.org/10.1016/j.ridd.2011.01.033
8	Kambas, A., Venetsanou, F., Kelaraki, D., & Karageorgopoulou, M. (2025). Group psychomotor therapy improves socio-emotional and motor competence of pre-school aged children, with and without attention deficit hyperactivity disorder. *Body, Movement and Dance in Psychotherapy*, 1-17. https://doi.org/10.1080/17432979.2025.2483278
9	Karanth, P., Shaista, S., & Srikanth, N. (2010). Efficacy of communication DEALL--an indigenous early intervention program for children with autism spectrum disorders. *Indian journal of pediatrics, 77*(9), 957-962. https://doi.org/10.1007/s12098-010-0144-8
10	Kennedy-Behr, A., Rodger, S., & Mickan, S. (2015). Play or hard work: Unpacking well-being at preschool. *Research in Developmental Disabilities*, *38*, 30-38. https://doi.org/10.1016/j.ridd.2014.12.003
11	Ketcheson, L., Hauck, J., & Ulrich, D. (2017). The effects of an early motor skill intervention on motor skills, levels of physical activity, and socialization in young children with autism spectrum disorder: A pilot study. *Autism, 21*(4), 481-492. https://doi.org/10.1177/1362361316650611
12	Ketcheson, L., Hauck, J. L., & Ulrich, D. (2018). The levels of physical activity and motor skills in young children with and without autism spectrum disorder, aged 2-5 years. *Autism, 22*(4), 414-423. https://doi.org/10.1177/1362361316683889
13	Ketcheson, L., Staples, K., Pitchford, E. A., & Loetzner, F. (2023). Promoting positive health outcomes in an urban community-based physical activity intervention for preschool aged children on the autism spectrum. *Journal of autism and developmental disorders*, *53*(2), 633-647. https://doi.org/10.1007/s10803-021-04871-7
14	Lakes, K. D., Abdullah, M. M., Youssef, J., Donnelly, J. H., Taylor-Lucas, C., Goldberg, W. A., Cooper, D., & Radom-Aizik, S. (2017). Assessing parent perceptions of physical activity in families

	of toddlers with neurodevelopmental disorders: The Parent Perceptions of Physical Activity Scale (PPPAS). *Pediatric Exercise Science, 29*(3), 396-407. https://doi.org/10.1123/pes.2016-0213
15	LaVesser, P., & Berg, C. (2011). Participation patterns in preschool children with an autism spectrum disorder. *OTJR: Occupation, Participation and Health, 31*(1), 33-39. https://doi.org/10.3928/15394492-20100823-01
16	Liberman, L., Ratzon, N., & Bart, O. (2013). The profile of performance skills and emotional factors in the context of participation among young children with developmental coordination disorder. *Research in Developmental Disabilities: A Multidisciplinary Journal, 34*(1), 87-94. https://doi.org/10.1016/j.ridd.2012.07.019
17	Lin, C.-Y., & Chang, Y.-M. (2015). Interactive augmented reality using Scratch 20 to improve physical activities for children with developmental disabilities. *Research in Developmental Disabilities, 37*, 1-8. https://doi.org/10.1016/j.ridd.2014.10.016
18	Medina-Mirapeix, F., Lillo-Navarro, C., Montilla-Herrador, J., Gacto-Sánchez, M., Franco-Sierra, M. Á., & Escolar-Reina, P. (2017). Predictors of parents' adherence to home exercise programs for children with developmental disabilities, regarding both exercise frequency and duration: A survey design. *European journal of physical and rehabilitation medicine, 53*(4), 545-555. https://doi.org/10.23736/S1973-9087.17.04464-1
19	Nelson, C., Paul, K., Johnston, S. S., & Kidder, J. E. (2017). Use of a creative dance intervention package to increase social engagement and play complexity of young children with autism spectrum disorder. *Education and Training in Autism and Developmental Disabilities, 52*(2), 170-185. https://www.jstor.org/stable/26420388
20	Salem, Y., Gropack, S. J., Coffin, D., & Godwin, E. M. (2012). Effectiveness of a low-cost virtual reality system for children with developmental delay: A preliminary randomised single-blind controlled trial. *Physiotherapy, 98*(3), 189-195. https://doi.org/10.1016/j.physio.2012.06.003
21	Sánchez, G. F. L., Williams, G., Aggio, D., Vicinanza, D., Stubbs, B., Kerr, C., Johnstone, J., Roberts, J., & Smith, L. (2017). Prospective associations between measures of gross and fine motor coordination in infants and objectively measured physical activity and sedentary behavior in childhood. *Medicine, 96*(46), e8424. https://doi.org/10.1097/MD.0000000000008424
22	Schenkelberg, M. A., McIver, K. L., Brown, W. H., & Pate, R. R. (2020). Preschool environmental influences on physical activity in children with disabilities. *Medicine & Science in Sports & Exercise, 52*(12), 2682-2689. https://doi.org/10.1249/MSS.0000000000002401
23	Takahashi, H., An, M., Matsumura, T., Seki, M., Ogawa, Y., Sasai, T., ... & Kato, T. (2023). Effectiveness of dance/movement therapy intervention for children with intellectual disability at an early childhood special education preschool. *American Journal of Dance Therapy*, *45*(1), 20-40. https://doi.org/10.1007/s10465-022-09356-3
24	Young, A., Healy, S., Silliman-French, L., & Brian, A. (2021). A pilot study of a parent-mediated, web-based motor skill intervention for children with down syndrome: Project skip. *Adapted Physical Activity Quarterly*, *38*(3), 452-473. https://doi.org/10.1123/apaq.2020-0171
25	Zachor, D. A., Vardi, S., Baron-Eitan, S., Brodai-Meir, I., Ginossar, N., & Ben-Itzchak, E. (2017). The effectiveness of an outdoor adventure programme for young children with autism spectrum disorder: A controlled study. *Developmental Medicine & Child Neurology*, *59*(5), 550-556. https://doi.org/10.1111/dmcn.13337
