# Supplementary material for: Physical Literacy and Physical Activity of Young Children with Developmental Disabilities: A Scoping Review
Source: Children (Basel). 2026 Apr 15;13(4):548. doi: 10.3390/children13040548 (PMC13115258; doi:10.3390/children13040548)
Supplement: Supplementary file 1 [file children-13-00548-s001.zip › Supplementary Table S4.pdf]

Supplementary Table S4. *Summary of selected studies*

References	Participants and country	Study aims
1. Bart *et al.* (2011). "How do young children with DCD participate and enjoy daily activities?"	63 children (M_{age} = 4.96 years, *SD* = 0.62; age range = 4.02 to 6.35 years) and their parents: 21 children with DCD; 21 children without DCD who were referred to occupational therapy due to mild developmental problems; 21 children from mainstream public kindergartens who did not require any developmental intervention.<br>Vancouver, Canada	To evaluate multidimensional aspects of participation amongst preschool children with and without DCD.
2. Becerra *et al.* (2021). "The effect of photographic activity schedules on moderate-to-vigorous physical activity in children with autism spectrum disorder."	3 children (1 boy and 2 girls with ASD; aged 4 years)<br>Utah, USA	To evaluate which environment (outdoor or indoor activities) produced the lowest percent of MVPA as recorded by the *Observational System for Recording Physical Activity in Children* and to assess the effectiveness of photographic activity schedules on engagement of MVPA in children diagnosed with ASD.
3. Draudvilienė, L., et al. (2024). "Two physiotherapy methods to improve the physical condition of children with autism spectrum disorder."	30 children (24 boys and 6 girls with ASD; M_{age} = 5.37, *SD* = .76; age 4-6 years old)<br>Basel, Switzerland.	To evaluate which method is more effective in improving balance, coordination, and motor skills; namely a physiotherapy program in a gym and games on a smart board with balance plates and an unstable base, to improve the physical condition of children with ASD; and to present which method motivates children more for physical activity
4. Fabrizi (2015). "Splashing our way to playfulness! An aquatic playgroup for young children with autism, a repeated measures design."	6 boys and 4 girls with ASD (age range = 29 to 36 months) and their caregivers<br>Florida, USA	To investigate the effectiveness of an aquatic playgroup on the playfulness of children, ages 2 to 3 with ASD.
5. Favazza *et al.* (2013). "Young athletes: A special olympics motor skill development program."	233 children (DD = 72%; ASD = 20%; communication disorder = 3 %; intellectual disability = 2%; other = 3%), 3- to 5- years old; 89 parents and 26 teachers.<br>Boston, USA	To examine the effectiveness of the Young Athletes program to promote motor development in preschool-aged children with disabilities.
6. Hastie *et al.* (2016). "An ecological analysis of a preschool mastery climate physical education programme."	13 children (11 boys and 2 girls; aged 4 years at the beginning of the programme) attendees at a day-care centre serving mostly African American children environmentally at risk for developmental delay and poor health.<br>Auburn, USA	To provide a microanalysis of life in a mastery climate which was grounded in the classroom ecology paradigm.

7. Jarus *et al.* (2011). "Participation patterns of school-aged children with and without DCD."	50 children (M_{age} = 6.07, *SD* = .61; 5–7 years old, 46 boys and 4 girls): 25 with DCD and 25 without DCD<br>Vancouver, Canada	To examine the participation patterns of children with and without DCD in their out-of-school-time activities, and to see whether there is a relationship between the children's motor abilities and their choices and participation.
8. Kambas, A., et al. (2025). "Group psychomotor therapy improves socio-emotional and motor competence of pre-school aged children, with and without attention deficit hyperactivity disorder."	54 children (31 boys, 23 girls; aged 61–69 months); 35 children (11 boys, 14 girls) were typically developing (NON-ADHD; M_{age} = 51.7 ± 3.09 months), while 19 children (12 boys, 7 girls) had ADHD (M_{age} = 50.62 ± 3.52 months)<br>Athens, Greece	To investigate the effects of group psychomotor therapy (GPT) on the socio-emotional and motor competence of children aged 5–6 years, both with and without ADHD, in a group setting, providing insights into its potential benefits.
9. Karanth *et al.* (2010). "Efficacy of communication DEALL--an indigenous early intervention program for children with autism spectrum disorders."	30 children with ASD including 21 boys and 9 girls (age range = 2.2 to 5.5 years) and their parents<br>Bangalore, India	To establish the efficacy of Communication DEALL, an indigenous early intervention program, in the management of children with ASD.
10. Kennedy-Behr, *et al.* (2015). "Play or hard work: Unpacking well-being at preschool."	63 preschool children aged 4 years 0 month to 6 years 11 months with (n = 32) and without (*n* = 31) probable DCD and their parents.<br>Munich, Germany	To examine the relationship between engagement in play and well-being for preschool children with and without DCD.
11. Ketcheson *et al.* (2017). "The effects of an early motor skill intervention on motor skills, levels of physical activity, and socialization in young children with autism spectrum disorder: A pilot study."	20 children with ASD aged 4–6 years: EG (*n* = 11) participated in an 8-week intervention; CG (*n* = 9) did not receive the intervention.<br>Southeast Michigan, USA	To measure the efficacy of an intensive motor skill intervention on motor skills, physical activity, and socialization in young children with ASD.
12. Ketcheson *et al.* (2018). "The levels of physical activity and motor skills in young children with and without autism spectrum disorder, aged 2-5 years."	53 children, including TD children (M_{age} = 42.5 ± 10.78 months, *n* = 19) and children with ASD (M_{age}= 47.42 ± 12.81 months, *n* = 34).<br>Southeast Michigan, USA	To examine relationships between the levels of physical activity and health outcomes.
13. Ketcheson *et al.* (2023). "Promoting positive health outcomes in an urban community-based physical activity intervention for preschool aged children on the autism spectrum."	25 children with ASD (M_{age} = 4.67; SD = 0.82 year; age range = 3 to 5 years; 18 boys [72%] and 7 girls [28%]).<br>Michigan, USA	To examine impact of a physical activity intervention on physical activity, fitness, and motor competence and to examine associations between motor behavior and ASD symptoms.
14. Lakes *et al.* (2017). "Assessing parent perceptions of physical activity in families of toddlers with	143 children (M_{age} = 31.65 months; 75% male) and their parents, 83% of the children received an ASD diagnosis.<br>California, USA	To examine a new tool (PPPAS = Parent Perceptions of Physical Activity Scale-Preschool) developed to study

neurodevelopmental disorders: The Parent Perceptions of Physical Activity Scale (PPPAS)."		parental perceptions of physical activity (PA) among parents of toddler and preschool age children.
15. LaVesser & Berg (2011). "Participation patterns in preschool children with an autism spectrum disorder."	144 children (M_{age} = 50 months; SD = 9.1, age range = 36 to 72 months; 113 boys [78.5%] and 31 girls [21.5%]) and their parents. 103 children with ASD (71.5%) and 41 children (28.5%) had no diagnosis by parent report.<br>Denver, USA	To compare participation patterns and barriers to participation in children with an ASD with those of children with no diagnosis.
16. Liberman *et al.* (2013). "The profile of performance skills and emotional factors in the context of participation among young children with developmental coordination disorder."	50 children (M_{age} = 5.29; *SD* = .42; 32 boys [64%] and 28 girls [36%], age range = 5 to 6.11 years) and their parents; 25 children diagnosed with DCD; 25 TD children<br>Tel Aviv, Israel	To look into the relations between participation and senses of coherence, effort and hope among children with DCD, in comparison to typically developed children.
17. Lin & Chang (2015). "Interactive augmented reality using Scratch 2.0 to improve physical activities for children with developmental disabilities."	3 children (2 girls and 1 boy; age range = 3-year 11-month to 6-year-old) with developmental disabilities<br>Taiwan city, Taiwan	To examine how to use an external webcam to detect movement and give real-time feedback to enhance the body strength of children with disabilities.
18. Medina-Mirapeix *et al.* (2017). "Predictors of parents' adherence to home exercise programs for children with developmental disabilities, regarding both exercise frequency and duration: A survey design."	219 parents of children with developmental disabilities receiving home exercise program-HEP (age range = 0.5 to 5 years; 46.6% were 2 years old or younger; boys = 63.9%)<br>Murcia, Spain	To examine whether the different behaviours of health professionals (e.g. physiotherapists), and the behaviour and social characteristics of parents determine rates of parental adherence to both the frequency per week, and duration per session, of HEP for children with developmental disabilities attending paediatric services in early intervention centres.
19. Nelson *et al.* (2017). "Use of a creative dance intervention package to increase social engagement and play complexity of young children with autism spectrum disorder."	3 ASD children; age range = 3 to 4 years<br>Utah, USA	To examine the effects of a strategy that utilized preferred play materials, antecedent creative dance activities, and priming of complex play with preferred play materials within dance activities on play behaviors of children with ASD during learning centers in inclusive preschool classrooms.
20. Salem *et al.* (2012). "Effectiveness of a low-cost virtual reality system for children with developmental delay: A preliminary randomised single-blind controlled trial."	40 children with developmental delay (M_{age} = 48.6 months; *SD* = 5.7 months; age range = 39 to 58 months; 22 boys [55%] and 18 girls [45%]); experimental (Wii) group (*n* = 20) and a control group (*n* = 20).<br>New York, USA	To determine the feasibility and preliminary effectiveness of a low-cost gaming system for young children with developmental delay.
21. Sánchez *et al.* (2017). "Prospective associations between measures of gross and fine motor coordination in infants and objectively measured physical	One parent (usually the child's mother) of 18 818 children at 9 months of age. Further surveys were administered at ages 3, 5, and 7 years.<br>Anglia Ruskin, United Kingdom	To investigate whether gross and fine motor delays in infants (aged 9 months) were associated with objective and self-reported activity levels later in childhood (aged 7 years).

activity and sedentary behavior in childhood."		
22. Schenkelberg *et al.* (2020). "Preschool environmental influences on physical activity in children with disabilities."	34 preschool-age children (M_{age} = 4.28; *SD* = 1.07; age range = 3 to 5 years; male = 64.7%) with developmental disabilities<br>Southeastern state, USA	To describe associations between physical and social environmental features of preschools and physical activity behaviors of young children with developmental disabilities.
23. Takahashi *et al.* (2023). "Effectiveness of Dance/Movement therapy intervention for children with intellectual disability at an early childhood special education preschool."	21 children with intellectual disability (16 boys, 5 girls; M_{age} = 46.1 ± 10.8 months; age range = 36 to 72 months)<br>Kyoto, Japan	To assess the effectiveness of DMT group sessions for children with ID aged 36 to 72 months as part of an early childhood special education preschool program.
24. Young *et al.* (2021). "A pilot study of a parent-mediated, web-based motor skill intervention for children with Down syndrome."	24 families with children diagnosed with Down syndrome (13 boys, 11 girls; age range = 3 to 7 years; M_{age} = 4.92; *SD* = 1.18)<br>Texas, USA	To examine the feasibility (i.e., participant engagement, parents' satisfaction, perceived strengths, and weaknesses) and preliminary effectiveness of a 6-week, parent-mediated, web-based intervention on the ball skills (i.e., catch, overhand throw) of children with DS.
25. Zachor *et al.* (2017). "The effectiveness of an outdoor adventure programme for young children with autism spectrum disorder: A controlled study."	51 children (40 males, 11 females; M_{age} = 5 years 4 months; *SD* = 11 months; age range = 3 years 4 months to 7 years 4 months) enrolled in ASD special education kindergartens. Intervention group (*n* = 30); Control group (*n* = 21)<br>Zerifin, Israel	To examine the effectiveness of an outdoor adventure programme in ASD children.

Note. M = mean; *SD* = standard deviation; ASD = autism spectrum disorder; DCD = developmental coordination disorder; DD = developmental disorder; DS = Down syndrome; DMT = dance movement therapy; ID = intellectual disabilities; TD = typical development; EG = experimental group; CG = control group; MVPA = moderate-to-vigorous physical activity
