# Supplementary material for: Physical Literacy and Physical Activity of Young Children with Developmental Disabilities: A Scoping Review
Source: Children (Basel). 2026 Apr 15;13(4):548. doi: 10.3390/children13040548 (PMC13115258; doi:10.3390/children13040548)
Supplement: Supplementary file 1 [file children-13-00548-s001.zip › Supplementary Table S5.pdf]

Supplementary Table S5. *Programs' description*

Reference	Aims	Duration	Description	Fidelity of implementation
1.Draudvilienė *et al.* (2024).	To evaluate which method is more effective in improving balance, coordination, and motor skills; and to present which method motivates children more for physical activity.	3 days/week for 5 weeks	*Schedule*: ---<br>*Facilitators*: Both physiotherapy programs were applied in the children's natural environment, an indoor gym. One of the researchers had already worked with autistic children for more than 3 years in the kindergarten. Some of the children participating in the study had known this person for at least one year. This person participated in all research stages. Therefore, before starting the procedural tests, each child was communicated with for about one week. This allowed the children to get used to the new person and facilitated the assessment of each child's perceptual abilities (listening and task performance).<br>*Activities:* Physical activities in the gym: The chosen physiotherapy program in a gym consists of simple exercises such as exercises on unstable surfaces, exercises with balls, throwing, catching, and hitting, as well as walking over obstacles to improve coordination. Smart board games: The smart board exercises were organized in such a way that the children had to reach the smart board by walking on an unstable surface to play on the board, and any touch or hand movement to the smart board was done while standing on the balance plates.	N/A
2. Fabrizi (2015)	To examine the effectiveness of the aquatic playgroup on the playfulness of young children with autism spectrum disorder (ASD), the caregiver's ability to promote play, and the supportiveness of a sensory-rich play environment located in the community.	40 min./week for 6 weeks	*Schedule*: Session (40 min total); Greeting (5 min); Transition into water (5 min); Group song and dance (5 min); Model/coach as group; Water skill and play object (15 min); Model/coach individual dyads and group; Transition out of water (5 min); Closure (5 min)<br>*Facilitators*: Four occupational therapy graduate students and one occupational therapist provided six intervention sessions. Lifeguards were provided by the aquatic center to ensure child and caregiver safety.<br>*Activities:* Modeling of playful behaviours using song and exaggerated gestures to encourage social participation. The use of objects allowed for coaching caregivers in water skills and play with caregiver and peers. Objects were also used as a visual cue to transition to the next part of the playgroup. Water activities represented an increasing demand for interaction within the aquatic environment. Children were able to wade through the water, crawl through tunnels, climb up stairs, slide, and splash—providing a range of sensory opportunities. Objects and skills demonstrated in previous sessions were also incorporated into subsequent sessions. Explore periods were used to coach caregivers in following the child's lead and interest, imitating actions, and setting up opportunities for social interactions and play through use of objects and people in the environment.	Intervention fidelity checklists were used to ensure procedural fidelity, and the intervention was manualized for future use.
3. Favazza *et al.* (2013)	To examine the benefits and impact of participation in the Young Athletes (YA)	30 min./day; 3 days/week for 8 weeks	*Schedule*: Each 30-minute lesson includes an opening motor movement song (4-5 minutes), motor games and activities (approximately 20 minutes), and closing (cool down) motor song (4-5 minutes).<br>*Facilitators*: Teachers and teacher assistants in classes assigned to the intervention group trained for 2 hours before implementation of the YA motor program.	Four indicators: attendance; Teacher Implementation

	motor program on the motor skills of preschool children.		*Activities:* The YA program consists of 24 comprehensive lessons, which include motor activities for foundational skills (visual tracking, motor imitation), walking and running, balance and jumping, trapping and catching, throwing, striking, and kicking to promote motor skill development of young children (less than 8-years-old) with disabilities through a series of motor play activities.	and Evaluation Log; Fidelity of Implementation Checklist; the YA Home Record.
4. Hastie *et al.* (2016)	To provide a microanalysis of life in a mastery climate grounded in the classroom ecology paradigm.	30 min./day; 2 days/week for 15 weeks	*Schedule*: Instruction and services from 7:30 am until 2:00 pm. The gross motor activity program was delivered 2 days a week (Tuesdays and Thursdays) in a mastery-motivational climate at the authors' university, and 3 days a week (Mondays, Wednesdays, and Fridays) in a free play environment at the day-care centre.<br>*Facilitators*: The teacher in this study was a faculty member at the university where the program took place. As a certified physical education teacher, she has extensive experience teaching in mastery climates and has conducted numerous studies investigating mastery-motivational climates with children aged 2 to 8 over the past 20 years.<br>*Activities:* individual and group activities in arts, crafts, reading, writing, arithmetic, and gross motor activity. Each session (30 min.) consisted of 6 to 8 stations in which children could practise various motor skills. Selected station activities were locomotor skills of running, galloping, hopping, leaping, horizontal jumping, sliding, as well as the object control skills of striking a stationary ball, dribbling a ball, kicking, catching, overhand throwing, and underhand rolling. Stations also promoted foundational abilities necessary to perform motor skills, such as leg and arm strength and core balance. Examples in these cases included climbing onto a tall mat and jumping off, walking on a balance beam or on turtle cones, rolling on tumbling mats, and crawling through tunnels to learn spatial awareness and how to manoeuvre the body and its parts. Hanging balloons were used to promote vertical jumping to subsequent leg strength and eye–hand coordination.	Two investigators analyzed eight 10-minute lesson segments (randomly selected): both agreed that these segments were in 100% compliance with the criteria.
5. Kambas *et al.* (2025).	To foster independence, responsibility, social skills, self-awareness, motor skills, self-esteem, empathy, trust, self-respect, and acceptance.	50 mins/day; 3 days/week for 6 months	*Schedule:* ---<br>*Facilitators:* Therapists adopt a subtle and 'invisible' role, allowing children to feel included and at ease, enabling better observation of each child's needs within the group dynamic. The group setting provides a natural social environment where children interact, face challenges, and learn to follow shared rules, which are co-created with the children. This dynamic is particularly beneficial for children facing the greatest challenges, as they can thrive in the supportive and inclusive nature of the group. Initial sessions provided individualised treatment within the group to ensure each participant could master personal objectives, regardless of their starting level.<br>*Activities:* Sessions consist of three components: (a) the awakening phase to foster group cohesion, (b) the therapeutic core to develop motor skills and address cognitive and socio-emotional aspects, and (c) relaxation rituals to conclude the session.	N/A
6. Karanth *et al.* (2010)	To establish the efficacy of Communication	3h/day; 5 days/week for 8 months	*Schedule*: ---<br>*Facilitators*: A multidisciplinary team of speech-language pathologists, occupational therapists, and developmental educators	N/A

	DEALL, an indigenous early intervention program, in the management of children with ASD.		*Activities:* ABA design; the skills the Communication-DEALL (Developmental Eclectic Approach to Language Learning) aimed to develop include Gross Motor skills (GM), Fine Motor skills (FM), Activities of Daily Living (ADL), Receptive Language (RL), Expressive Language (EL), Cognitive Skills (CS), Social Skills (SS) and Emotional Skills (ES). However, the specific activities during therapy are not mentioned in the article. For further details, see: https://www.communicationdeall.com/	
7. Ketcheson *et al*. (2017)	To measure the efficacy of a motor skill intervention on motor skills and levels of PA, implementing Classroom Pivotal Response Teaching (CPRT) as a framework for instruction in preschool-aged children with ASD.	4h/day; 5 days/week for 8 weeks	*Schedule:* the summer months<br>*Facilitators:* The principal investigator of this intervention had 10 years of combined teaching and research experience related to early motor behaviour programming. All research assistants were undergraduate students who had previously worked with children with disabilities and were interested in pursuing a graduate degree in pediatrics or a related field. Parents, peers or service providers (occupational therapist, physical therapist, adapted physical education teacher) act as the principal intervention agent.<br>*Activities:* Learning opportunities in CPRT can occur within the child's natural environment. There are eight key components to the CPRT program grouped by antecedent (e.g., student attention, clear and appropriate language, easy and difficult tasks, shared control, and multiple cues) and consequence strategies (direct reinforcement, contingent consequence, and reinforcement of attempts). Specifically, children participated in free play activities (indoor gym), in 1:1 direct instruction and in small group activities developing locomotor or object control skills on the soccer field.	During the first week, all research assistants were evaluated for fidelity (minimum of 80%). Afterwards, it was assessed on a bi-weekly basis.
8. Ketcheson *et al*. (2023)	To examine the impact of a physical activity intervention on physical activity, fitness, and motor competence of children with ASD.	1h/week for 12 weeks	*Schedule:* The intervention followed a consistent routine – warm up (5 minutes), task card; review of previously acquired skills (10 minutes), introduction of two new skills (3 minutes), introduction of small group activity (2 minutes), small group activity (15 minutes), water break (3 minutes), introduction of large group activity (2 minutes), large group activity (15 minutes), review (2 minutes), and cool down (3 minutes).<br>*Facilitators:* The intervention included two different types of instructors; coaches who worked in 1:1 dyad with research participants, and lead teachers.<br>*Activities:* The small group activity was a goal-oriented task that encouraged participation from all group participants to complete. The large group activity consisted of a time-oriented task.	N/A
9. Nelson *et al*. (2017)	To evaluate the effectiveness of an intervention package that included priming	10 min./day; 3-4 days/week for 10 weeks	*Schedule:* 10-minute creative dance activity during the regular circle time<br>*Facilitators:* A licensed early childhood special education teacher, who was a researcher on this project, provided intervention for all participants. The facilitator had a graduate degree in early childhood special education and was a licensed and experienced	Calculated by dividing the number of interventionist

	of social play and more complex use of preferred toys within creative dance activities on increasing quantity and quality of engaged play of young children with ASD within subsequent free-choice play or "learning" centers.		teacher of children with ASD. In addition, she had extensive experience as a creative dance educator with young children with a variety of disabilities.<br>*Activities:* The classes had approximately 16 children each and most of the children were typically developing. Dance activities included both time on the circle time rug and time engaging in large motor activities, moving around the classroom. Dance activities varied each day but unfolded in a predictable sequence with an opening hello song and a warm-up movement activity followed by dance activities that involved movement throughout the classroom, and finally a closure activity with a slow tempo, transition back to the rug, and a goodbye activity as materials were put away. The facilitator varied the tempo, rhythm, and intensity during each dance activity based on the participating child's reactions and perceived emotional regulation. After the dance session, the three identified preferred objects were placed in three different play centers and highlighted to all children in the class. Instruction on use of preferred objects was not provided and their use was not modeled or primed during the dance activity.	behaviours exhibited by the number of planned interventionist behaviours and multiplying by 100.
10. Salem *et al.* (2012)	To demonstrate the feasibility, safety and effectiveness of using a low-cost commercially available gaming system in the rehabilitation of children with developmental delay.	30 min./day; 2 days/week for 10 weeks	*Schedule:* ---<br>*Facilitators:* A licensed physical therapist and paediatric certified clinical specialist with 17 years of experience.<br>*Activities:* The physical activities in these games include motor tasks that involve a wide range of sensory feedback; adjustable movement amplitudes, speed and precision levels; and incorporation of a variety of visual–spatial, cognitive and attention tasks. The Wii training program focused on children's balance, walking, strength, weight bearing and aerobics. Training activities were selected to challenge the participant's balance, strength and walking. A personal profile was created for each child, and the selection of games and activities were individualized for each child based on their interests, functional limitations, and abilities. In each training session, the child played games such as strength (Lunges and Single Leg Stance), balance (Soccer Heading, Penguin Slide and Tightrope) and aerobics (Basic Run, Hula Hoop and Basic Step). Each session was concluded by playing of one of the following games based on the interest of the child: baseball, boxing or bowling. A therapist from one of the clinical sites supervised each training session.	N/A
11. Takahashi *et al.* (2023)	To assess the effectiveness of Dance Movement Therapy group sessions for children with intellectual disabilities as part of	60 min./week for 10 weeks	*Schedule:* Each session includes a warm up, a first main activity, a second main activity, and a cool down.<br>*Facilitators:* A dance/movement therapist certified by the American Dance Therapy Association and Japanese Dance Therapy Association was the group leader, and three to four teachers from the preschool provided support to the group as co-group leaders.<br>*Activities*: Mirroring the therapist's and others' movements; synchronizing movements with the therapist, co-leaders, and other participants; moving their own inner image as an authentic movement intervention, and applying effort involved in Laban Movement Analysis.	N/A

	an early childhood special education preschool program.			
12. Young *et al. (*2021)	To examine the feasibility (i.e., participant engagement, parents' satisfaction, perceived strengths, and weaknesses) and preliminary effectiveness of a 6-week, parent-mediated, web-based intervention on the ball skills (i.e., catch, overhand throw) of children with Down syndrome.	≈ 20 min./day; 3 days/week for 6 weeks	*Schedule:* The summer months<br>*Facilitators:* Parents supported by a Facebook platform, instructional videos, activity ideas, weekly reminders, and posting of photos/videos of other children.<br>*Activities:* Project SKIP consisted of four intervention components that targeted one or more constructs of the social cognitive theory and included: (a) motor learning activities/instructions, (b) social prompts (once per week, social prompts are provided as posts to the group to stimulate interaction between parent participations), (c) reflection posts (once per week, participants are asked to reflect on how many days during the week their children completed Project SKIP activities), and (d) motivational posts (once per week, participants received a post aimed at enhancing their beliefs about the importance of fundamental motor skills for their child's development). Twice weekly, investigators posted motor learning activities/instructions. The posts focused on target skills (e.g., hands ready), teaching cues for the target skills (e.g., point to target), practice activity ideas (e.g., hot potato), and how to provide specific feedback (e.g., "I like how you used your hands to catch").	Parents reported on time spent by their child in Project SKIP activities.
13. Zachor *et al.* (2017)	To examine the effectiveness of an outdoor adventure program in children with ASD.	30 min./day; 13 weeks	*Schedule:* ---<br>*Facilitators:* The outdoor adventure program staff, including a senior guide and two field instructors, led the activities. The senior guide defined the research rationale and the characteristics of the intervention and gave professional feedback. Every field instructor underwent an extensive internal training program provided by the organization.<br>*Activities:* Each session took place in urban parks near the participants' kindergartens and started with an opening song, after which the children began using the devices (e.g. two-way climbing rope ladder, rope elevator, rope bridge, hammock, and rope swing), moving from one to another throughout the session. At the end of the session, everyone gathered for a brief closing meeting in which the children were asked, "how was it?" and "what activities did you enjoy?" Then the group sang a closing song.	The senior guide's involvement in all research stages and the extensive training program ensured fidelity of implementation of the intervention.
